# Predicting incident dementia in community-dwelling older adults using primary and secondary care data from electronic health records

**DOI:** 10.1093/braincomms/fcae469

**Published:** 2024-12-24

**Authors:** Konstantin Georgiev, Yiqing Wang, Andrew Conkie, Annie Sinclair, Vyron Christodoulou, Saleh Seyedzadeh, Malcolm Price, Ann Wales, Nicholas L Mills, Susan D Shenkin, Joanne McPeake, Jacques D Fleuriot, Atul Anand

**Affiliations:** BHF Centre for Cardiovascular Science, Queen's Medical Research Institute, University of Edinburgh, Edinburgh EH16 4TJ, UK; Red Star, Glasgow G64 2BS, UK; BHF Centre for Cardiovascular Science, Queen's Medical Research Institute, University of Edinburgh, Edinburgh EH16 4TJ, UK; Red Star, Glasgow G64 2BS, UK; Red Star, Glasgow G64 2BS, UK; The Data Lab Innovation Centre, Bayes Centre, University of Edinburgh, Edinburgh EH8 9BT, UK; The Data Lab Innovation Centre, Bayes Centre, University of Edinburgh, Edinburgh EH8 9BT, UK; The Data Lab Innovation Centre, Bayes Centre, University of Edinburgh, Edinburgh EH8 9BT, UK; Digital Health & Care Innovation Centre, University of Strathclyde, Glasgow G1 1RD, UK; BHF Centre for Cardiovascular Science, Queen's Medical Research Institute, University of Edinburgh, Edinburgh EH16 4TJ, UK; Ageing and Health Research Group and Advanced Care Research Centre, Usher Institute, Edinburgh BioQuarter, University of Edinburgh, Edinburgh EH16 4UX, UK; The Healthcare Improvement Studies Institute, Department of Public Health and Primary Care, University of Cambridge, Cambridge CB1 8RN, UK; Artificial Intelligence and its Applications Institute, School of Informatics, Newington, University of Edinburgh, Edinburgh EH8 9AB, UK; BHF Centre for Cardiovascular Science, Queen's Medical Research Institute, University of Edinburgh, Edinburgh EH16 4TJ, UK

**Keywords:** primary prevention, health services, machine learning, geriatric care, risk identification

## Abstract

Predicting risk of future dementia is essential for primary prevention strategies, particularly in the era of novel immunotherapies. However, few studies have developed population-level prediction models using existing routine healthcare data. In this longitudinal retrospective cohort study, we predicted incident dementia using primary and secondary care health records at 5, 10 and 13 years in 144 113 Scottish older adults who were dementia-free prior to 1st April 2009. Gradient-boosting (XGBoost) prediction models were trained on two feature subsets: data-driven (using all 171 extracted variables) and clinically supervised (22 curated variables). We used a random-stratified internal validation set to rank top predictors in each model, assessing performance stratified by age and socioeconomic deprivation. Predictions were stratified into 10 equally sized risk deciles and ranked by response rate. Over 13 years of follow-up, 11 143 (8%) patients developed dementia. The data-driven models achieved marginally better precision-recall area-under-the-curve scores of 0.18, 0.26 and 0.30 compared to clinically supervised models with scores of 0.17, 0.27 and 0.29 for incident dementia at 5, 10 and 13 years, respectively. The clinically supervised model achieved comparable specificity 0.88 [95% confidence interval (CI) 0.87–0.88] and sensitivity (0.55, 95% CI 0.53–0.57) to the data-driven model for prediction at 13 years. The most important model features were age, deprivation and frailty, measured by a modified electronic frailty index excluding known cognitive deficits. Model precision was consistent across socioeconomic deprivation quintiles but lower in younger-onset (<70 years) dementia cases. At 13 years, dementia was diagnosed in 32% of the population classified as highest risk with 40% of individuals in this group below the age of 80. Personalized estimates of future dementia risk from routinely collected healthcare data could influence risk factor modification and help to target brain imaging and novel immunotherapies in selected individuals with pre-symptomatic disease.

## Teaser

Georgiev *et al*. use a machine learning approach to predict dementia risk in community-dwelling adults over 50 years old in Scotland. In the model’s highest risk decile, one in three people developed dementia over the following 13 years. These models could inform risk factor modification to improve long-term brain health.

## Graphical Abstract

**Graphical abstract:**
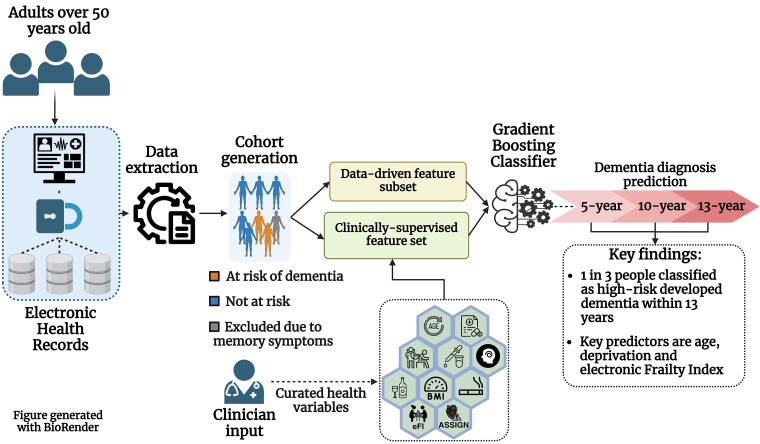

## Introduction

Over 800 000 older people are currently living with dementia in the UK and millions more worldwide. Within 25 years, this number is projected to double, with an average 45% increase in those aged 65 and over.^1,2^ Dementia-related costs are substantial, estimated at £26.3 billion annually in the UK,^3,4^ and around $355 billion across the USA for long-term health and care services.^5^ Recent trials of novel monoclonal antibody immunotherapy against amyloid deposition have demonstrated the potential to slow disease progression.^6,7^ However, these trials recruited participants experiencing very early cognitive impairment with eligibility confirmed by PET imaging or cerebrospinal fluid sampling, neither of which are routinely offered in clinical practice. Even in those with positive amyloid imaging, only a minority would be likely to meet trial eligibility criteria.^8^ If these drugs gain regulatory approval, there will be unprecedented pressures on already constrained diagnostic resources. As such, there is an urgent need to target imaging in pre-symptomatic individuals with the highest probability of future dementia.

Routinely collected electronic health record (EHR) data contain relevant information on dementia risk factors, such as those identified by the Lancet Commission.^9^ Studies suggest that signs of cognitive impairment and progressive neurodegeneration can occur up to 9 years prior to diagnosis.^10^ Data-driven studies have the potential to refine our understanding of future dementia risk, analogous to the approach adopted for proactive cardiovascular risk screening to target interventions such as lipid-lowering therapy.^11-13^ Aside from novel therapies, personalized brain health estimates could influence better risk factor control through supported individual lifestyle modifications.

Several studies have used machine learning approaches to predict future dementia, but these frequently use selective populations such as those undergoing specialized imaging,^14,15^ or where symptom concerns have already prompted memory clinic referral,^16,17^ or where detailed genetic profiling has been completed.^18,19^ While such approaches may have value in supporting efficient dementia diagnosis, they are not appropriate for pre-symptomatic risk identification across the whole population. Some EHR data models have recently been published.^20,21^ While these present an advancement in this field, limitations are still apparent, either in the observation periods or the lack of relevant linked data for socioeconomic status, lifestyle risk factors, and relevant frailty markers.

Our aim was to evaluate the utility of machine learning models developed using comprehensive linked routine primary and secondary care data to predict future dementia diagnosis. We report risk estimates of incident dementia at 5, 10 and 13 years across a large, unrestricted adult population in Scotland.

## Materials and methods

This study was performed with the delegated approval of the local Research Ethics Committee and Caldicott Guardian. All data were collected from EHRs and national registries previously de-identified by the DataLoch service (Edinburgh, UK) and analysed in a Secure Data Environment. Individual consent was not required for this study.

### Study design and participants

In this longitudinal retrospective cohort study, we included all older adults (50–102 years old) registered with a research-linked general practice in a regional health board in South East Scotland. This includes 90% of all general practices and covers a population of approximately 900 000 people of all ages. Only individuals who were alive without a recorded diagnosis of dementia in either the primary or secondary care electronic patient record systems on 1st April 2009 were eligible. Patient follow-up continued until 1st April 2023. Individuals with prior outpatient old age psychiatry clinic attendance for dementia assessment and diagnosis, or any of 111 primary care codes related to ‘memory and cognitive problems’ were excluded. We defined an observation window from 1st April 2009 to 1st April 2010, excluding individuals who were diagnosed with dementia or died in this period.

### Data sources

**Figure 1:**
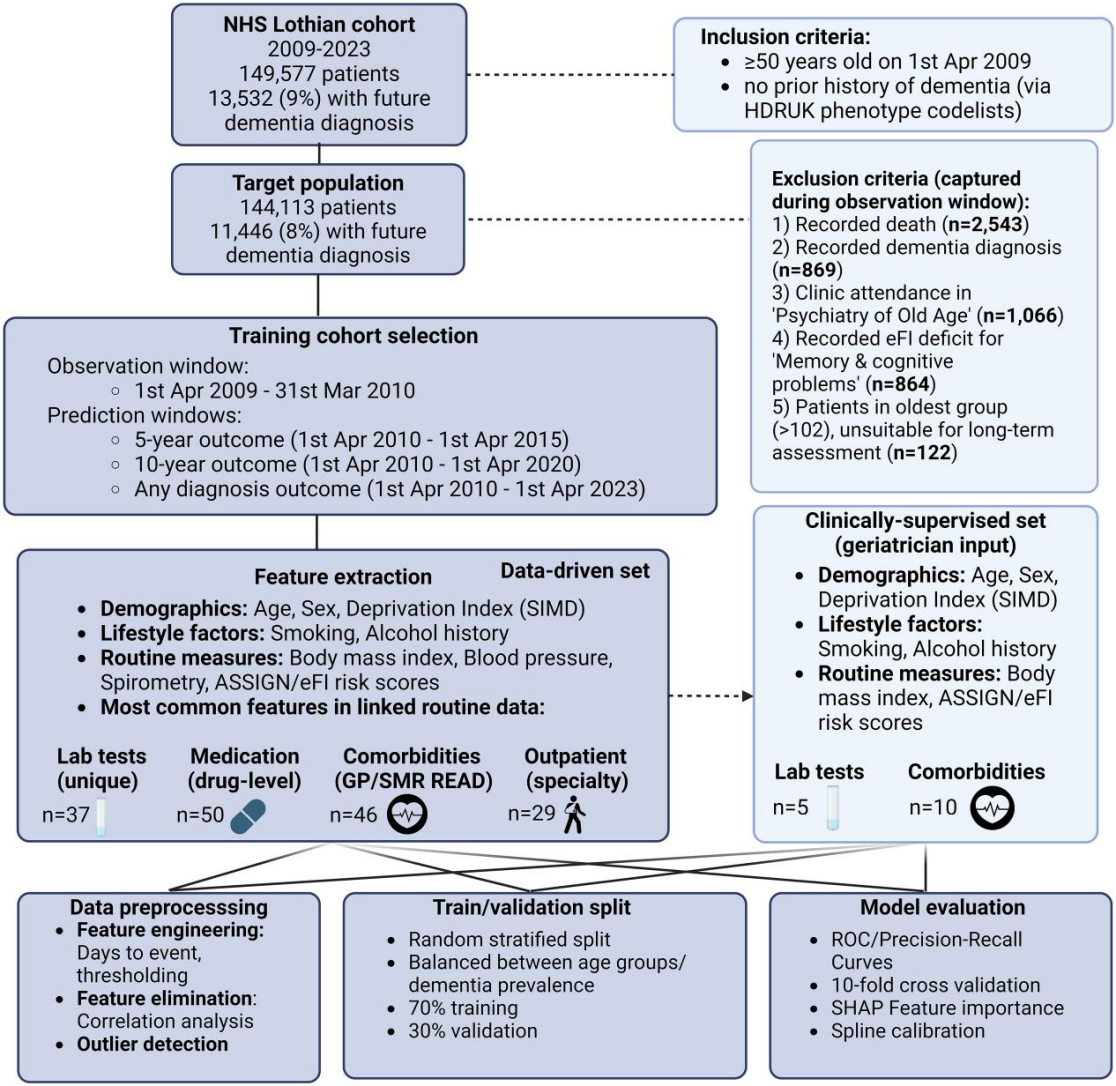
**Pipeline chart summarizing the data collection, preprocessing, model training workflow and overview of the data-driven and clinically supervised feature subsets**. SIMD, Scottish Index for Multiple Deprivation; ASSIGN, cardiovascular risk calculator validated in Scottish populations (Scottish Heart Health Extended Cohort); SHAP, SHapley Additive exPlanations framework for feature importance analysis; ROC, receiver operating characteristic; eFI, electronic Frailty Index, cumulative frailty-related deficit measure, modified to exclude memory and cognitive problems. Figure generated with BioRender.

A summary of the data flows in this study is presented in Fig. 1. Common comorbidities were defined using HDR UK CALIBER phenotype codelists^22^ for the presence of relevant codes acquired prior to or during the observation window in either GP records (Read version 2), or hospital (ICD-10 codes) using the Scottish Morbidity Records (SMR). We included information from outpatient clinic attendances (SMR00), acute inpatient episodes (SMR01) and acute mental health admissions (SMR04). We used the Scottish Index of Multiple Deprivation (SIMD) to stratify individuals across quintiles of relative socioeconomic deprivation.^23^ The cause of any deaths were identified from ICD-10 coded certification using linkage with the National Records of Scotland. Medication history was collected using a 6-month lookback window for the 50 most prescribed medications within the Scottish Prescribing Information System (Public Health Scotland), which contains records of all non-hospital dispensed prescriptions. Laboratory data for common haematology and biochemistry tests requested from either community or hospital settings were extracted from the local EHR system (TrakCare, InterSystems, MA, USA). Where completed in primary or secondary care, we extracted relevant coded records of lifestyle risk factors [alcohol, body mass index (BMI), smoking status] and measures of blood pressure and lung function (spirometry measures). The models also included two routinely recorded risk scores from primary care, representing cumulative deficit scores of frailty [electronic Frailty Index (eFI)]^24^ and 10-year cardiovascular disease risk (ASSIGN score).^25^ The eFI was modified to exclude prior reported memory or cognitive problems and was therefore calculated as a modified eFI using 35 deficits. The last valid record within the observation window was used for model development in all cases.

### Outcomes and prediction windows

The primary outcome was defined via a new dementia code in either primary, secondary or death records within 5, 10 or 13-year prediction windows starting from the index prediction date of 1st April 2010. We used the HDR UK CALIBER dementia phenotype,^22^ combining all dementia subtype codes. We additionally performed a sensitivity analysis for the more specific outcome of an Alzheimer's Disease-related Dementia (ADRD) diagnosis. All-cause mortality was a secondary outcome.

### Model setup and data cleaning

**Figure 2:**
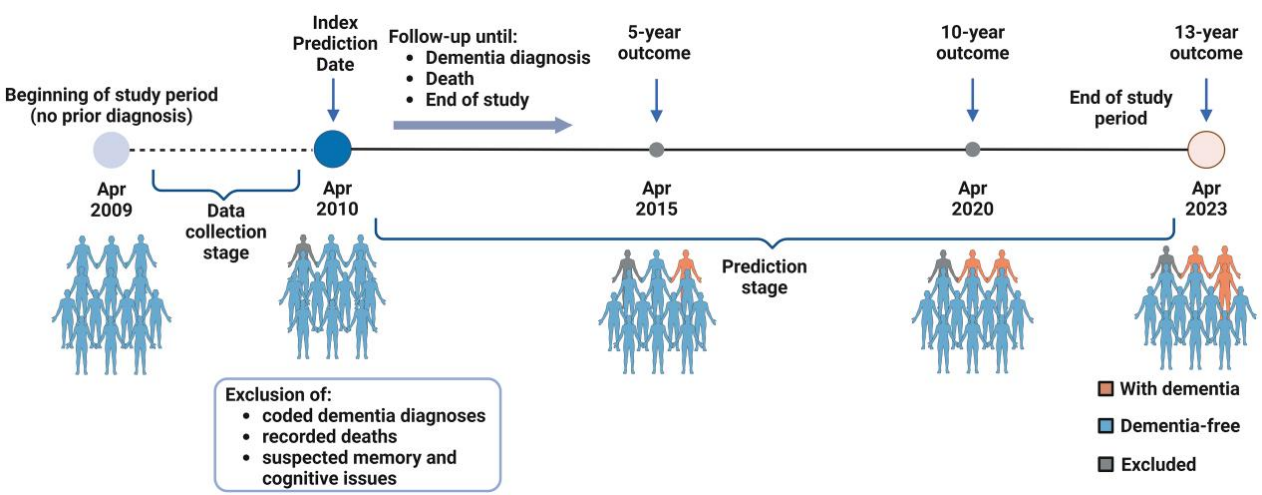
**Timeline flow diagram detailing the data collection and prediction windows for incident dementia relative to follow-up time**. Individuals with a recorded dementia diagnosis, death or suspected memory problems via clinic attendance or eFI coding were excluded from the training set. Figure generated with BioRender.

We undertook both a data-driven and a clinically supervised approach, creating two model variations per outcome. In the data-driven approach, we used the complete set of available routine data, totalling 219 continuous and 92 categorical variables for training (Fig. 1). Further details on these variables, the use of thresholds and temporal criteria are described in Supplementary Table 1. Details regarding patient follow-up, including observation and prediction windows across the three dementia outcomes are described in Fig. 2. In the clinically supervised approach, candidate features were selected using clinical input (by author A.A.). This included modifiable and non-modifiable risk factors known to impact dementia risk in the literature, with clinical relevance for early dementia screening.^9^ The complete list of 22 curated features included age, sex, SIMD (quintiles of multiple deprivation estimated across seven resource or income-based domains),^26^ alcohol and smoking history, BMI, modified eFI (EHR-based cumulative markers of frailty status)^24^ and ASSIGN (cardiovascular disease risk score incorporating SIMD)^25^ risk scores, selected blood tests (HDL/LDL/total cholesterol, triglycerides, glycosylated haemoglobin [HbA1c]) and long-term conditions from primary and secondary health records (atrial fibrillation, hearing loss, heart failure, ischaemic heart disease, hypertension, stroke, peripheral vascular disease, diabetes, obesity, alcohol and substance misuse). The model hyperparameters were fine-tuned using a cross-validated grid search strategy, targeting the 13-year outcomes (Supplementary Table 2). Models were developed using Python version 3.10.12, using the ‘xgboost’ package (version 2.0.3) for training and ‘scikit-learn’ (version 1.3.2) for evaluation and calibration procedures.

We employed additional data cleaning to remove sparse features with <1% completeness within the observation window. We removed samples containing any outlier measurements (<0.5 or >99.5 percentile of the dataset). Correlated features were removed when Pearson’s correlation coefficient was over 0.9, prioritizing the retention of continuous variables over defined categorical or temporal variables of the same nature.

### Training and validation procedures

We used an established ensemble model with gradient-boosted trees (XGBoost) to develop the dementia incidence and all-cause mortality models.^27^ We additionally tested performance on any future dementia diagnosis using other linear and non-linear estimators (logistic regression, naïve Bayes, decision trees and random forests). We performed a random stratified split (70% for training and 30% for validation), balancing for age, dementia incidence and all-cause mortality rates between the two sets. At the evaluation stage, we measured the receiver operating characteristic area-under-the-curve (*ROC-AUC*) and the precision-recall area-under-the-curve (*PR-AUC*). We measured the positive predictive value (*PPV*), negative predictive value (*NPV*), *Sensitivity* and *Specificity* through thresholding based on the maximum *F1-Score* achieved for the positive class per outcome. In the presence of high class imbalance between dementia and dementia-free cases, *ROC-AUC* can produce falsely elevated estimates, undermining the impact of the *PPV* score. Meanwhile, the *PR-AUC* provides a relative measure of trade-off between *PPV* and *Sensitivity* compared to baseline dementia risk. A higher score than the baseline disease prevalence is treated as ‘better than random choice’. We employed a *post hoc* calibration technique using cubic splines to normalize the probability distribution and re-evaluate the classifier.^28^ At the calibration stage, a further 30% of the training set was held out to fit the spline model and re-calibrate the probability scores within the internal validation set. The evaluation measures were reported post-calibration. A stratified 10-fold cross-validation strategy was used as an additional procedure to validate both the *PR-AUC* and *ROC-AUC* on random data partitions. The 95% confidence intervals (95% CI) for these values were generated using the DeLong method, optimized for large sample sizes using linearithmic weights.^29^ Stratified analyses were conducted, evaluating potential imbalances in classification performance across age groups and SIMD quintiles.

### Statistical analysis

Baseline differences in characteristics from the observation window between patients with and without a future dementia diagnosis are reported using the clinically supervised feature set. Continuous variables were measured using the Kruskal–Wallis test (for non-normal distributions), while categorical variables were reported using Pearson’s chi-squared test, where significance was assumed at *P* < 0.001. An unadjusted multivariate Cox Proportional Hazards model was used to test the significance of the clinically supervised features in relation to the timing of diagnosis. We ranked the top predictors in both the clinically supervised and data-driven models using the SHAP framework (Shapley Additive eXplanations)^30^ and the calibrated probability scores. The estimated patient-level Shapley values on the internal validation set were summarized in density plots, highlighting the observations contributing to increased (red) and decreased risk (blue). To perform risk stratification, we used quantile-based discretization on the validation set's sorted and calibrated probability scores to generate 10 equally sized risk groups. The response rate (% observed dementia diagnoses) and the age distribution across each model subset were then reported.

## Results

### Cohort summary

The cohort included 144 113 individuals, of whom 11 143 (8%) developed dementia during the 13-year prediction window. Baseline differences between the people who did and did not develop dementia are shown in Table 1. Those individuals who developed dementia were older at baseline [75 (69–80) versus 60 (55–69) years, *P* < 0.001] and more likely to be female (62% versus 51%, *P* < 0.001). The distribution of socioeconomic deprivation was similar between groups, but records were more complete in people with a diagnosis. In those who went on to develop dementia, rates of recorded smoking, high alcohol consumption and BMI measures were lower within the observation window when compared to those who remained free from dementia. However, the ASSIGN and modified eFI risk scores were higher, although the ASSIGN score was only recorded in 3% of all participants. In most clinically curated variables, completeness rates were higher in the dementia group in the early study years, but lower closer to the end of follow-up (Supplementary Fig. 1).

**Table 1:** Patient characteristics at baseline grouped by any future coded incident dementia

Characteristics	Missing	All (*n* = 144 113)	No dementia (*n* = 132 670)	Incident Dementia (*n* = 11 143)	*P*
Age (median, IQR)	0	61 (55–70)	60 (55–69)	75 (69–80)	<0.001
Sex	0				<0.001
Male		68 839 (48%)	64 481 (49%)	4358 (38%)	
Female		75 274 (52%)	68 189 (51%)	7085 (62%)	
SIMD in quintiles	32 843		31 732 (24%)	1111 (10%)	<0.001
1 (most deprived)		13 194 (9%)	11 938 (9%)	1256 (11%)	
2		22 070 (15%)	19 708 (15%)	2362 (21%)	
3		16 932 (12%)	15 335 (12%)	1597 (14%)	
4		20 272 (14%)	18 400 (14%)	1872 (16%)	
5 (least deprived)		38 802 (27%)	35 557 (27%)	3245 (28%)	
Lifestyle and medical risk factors					
Smoking (current)	22 799	26 303 (18%)	24 831 (19%)	1472 (13%)	<0.001
Alcohol (high consumption >6 u/day)	51 170	828 (0.6%)	795 (0.6%)	33 (0.3%)	<0.001
BMI (median, IQR)	40 353	26.60 (23.80–30.30)	26.70 (23.80–30.30)	26.40 (23.60–29.70)	<0.001
ASSIGN score (median, IQR)	140 068	13 (7–21)	13 (7–20)	21 (12–33)	<0.001
Modified eFI score (median–IQR)^a^	42 689	0.06 (0.03–0.11)	0.06 (0.03–0.11)	0.11 (0.06–0.17)	<0.001
Medical condition history	0				
Atrial fibrillation		6447 (5%)	5316 (4%)	1131 (10%)	<0.001
Ischaemic heart disease		13 655 (10%)	11 567 (9%)	2088 (18%)	<0.001
Heart failure		4305 (3%)	3652 (3%)	653 (6%)	<0.001
Hypertension		48 117 (33%)	41 857 (32%)	6260 (55%)	<0.001
Stroke		5464 (4%)	4473 (3%)	991 (9%)	<0.001
Peripheral vascular disease		3692 (3%)	3131 (2%)	561 (5%)	<0.001
Diabetes		12 278 (9%)	10 736 (8%)	1542 (14%)	<0.001
Obesity		18 266 (13%)	16 678 (13%)	1588 (14%)	<0.001
Alcohol/substance misuse		8176 (6%)	7606 (6%)	570 (6%)	0.001
Hearing loss		10 293 (7%)	8615 (7%)	1678 (15%)	<0.001
Lab tests (median, IQR)					
Total cholesterol (mmol/L)	118 301	4.6 (3.9–5.5)	4.6 (3.9–5.5)	4.5 (3.8–5.3)	<0.001
Triglycerides (mmol/L)	126 196	1.6 (1.1–2.3)	1.6 (1.2–2.3)	1.5 (1.1–2.1)	<0.001
HbA1c (mmol/mol)	138 004	53 (44–64)	53 (44–65)	52 (44–62)	0.118
Cholesterol ratio (Total/HDL, mg/dL)	127 734	3.5 (2.9–4.3)	3.6 (2.9–4.4)	3.3 (2.8–4.0)	<0.001
All-cause mortality	0	40 074 (28%)	32 107 (24%)	7967 (70%)	<0.001

Values are in proportion of patients (%) unless stated otherwise. Unadjusted *P*-values for continuous variables were generated using the Kruskal-Wallis H test, while the chi-squared test was used for categorical variables. SIMD, Scottish Index for Multiple Deprivation; ASSIGN, cardiovascular risk calculator validated in Scottish populations (Scottish Heart Health Extended Cohort); eFI (electronic Frailty Index), cumulative frailty-related deficit measure.

^a^Excludes deficits coded as ‘Memory & Cognitive problems’ from the estimation.

All clinically supervised comorbidities were more frequently observed in the group who developed dementia, except for prior alcohol or substance misuse. By the end of the study period, all-cause mortality rates were significantly higher in those who developed dementia (70% versus 24% in non-dementia cases, *P<* 0.001). Median observation time until death or end of study period was 125 (84–156) months in those who developed dementia, compared to 156 (156–156) months in those who did not (*P<* 0.001). However, in patients who died (*n* = 40 074), the observation period was longer in those with future dementia diagnosis [102 (68–128) versus 81 (40, 120) months without dementia, *P<* 0.001]. The rate of newly coded diagnoses in the routine data fluctuated over the years, with a notable drop following the COVID-19 pandemic in 2020 and a slower recovery in subsequent years (Supplementary Fig. 2A). Most participants (76% mean) had their index diagnosis coded within primary care (Supplementary Fig. 2B), although this could follow correspondence from a specialist outpatient clinic making the diagnosis. The mean age at diagnosis was 82 ± 7 years, and most dementia diagnoses were made in the 80–89 years old group (Supplementary Fig. 2C) after a median period of 78 (40–114) months from the index prediction date. There was non-specific subtype coding in 48% of dementia diagnoses, while 36% were ADRD-coded (Supplementary Fig. 2D). The linear Cox regression model (excluding age) suggested that modified eFI >0.05 (the equivalent of two or more non-cognitive deficits) had the highest positive association with the timing of diagnosis, and most curated risk factors significantly contributed to a future diagnosis (Supplementary Fig. 3).

After performing a stratified random split to generate the training (*n* = 101 286) and validation (*n* = 43 409) sets, the samples were fully balanced by age group, mortality and dementia incidence rates (Supplementary Table 3). The incidence at 5-, 10- and 13-year prediction windows was 3, 6 and 8% for any dementia diagnosis, 1, 2 and 3% for ADRD, and 9, 20 and 28% for all-cause mortality, respectively.

### Performance summary

Table 2 shows the performance of both the data-driven and clinically supervised models, estimated after calibration. Overall, the data-driven model performed marginally better than the model that used a clinically supervised subset of features. For the data-driven model, the ROC-AUC scores had similar discrimination for dementia [0.89 (0.88–0.89) at 5 years, 0.87 (0.86–0.87) at 10 years and 0.85 (0.84–0.85) at 13 years] and mortality [0.89 (0.89–0.90) at 5 years, 0.89 (0.88–0.89) at 10 years and 0.88 (0.88–0.89) at 13 years]. Using the *PR-AUC* and *F1-score* thresholded scores (*PPV*, *NPV*, *Sensitivity* and *Specificity*) to assess discrimination in detected cases, we observed that model performance improved as the prediction windows widened for both dementia [*PR-AUC* of 0.18 (0.13–0.23) at 5 years, 0.28 (0.24–0.32) at 10 years and 0.30 (0.26–0.34) at 13 years] and all-cause mortality [*PR-AUC* of 0.55 (0.51–0.59) at 5 years, 0.73 (0.70, 0.75) at 10 years and 0.79 (0.77, 0.81) at 13 years]. In this case, precision was limited among the dementia incidence models (0.14, 0.26 and 0.30 versus 0.54, 0.65 and 0.70 in all-cause mortality models at 5, 10 and 13 years, respectively). The *NPV* was more robust, consistent with the relatively low dementia incidence (0.99, 0.97 and 0.96 at 5, 10 and 13 years, respectively). Sensitivity for all-cause death improved with the longer prediction window (0.54, 0.67 and 0.72 at 5, 10 and 13 years, respectively) but worsened for dementia (0.76, 0.58 and 0.53 at 5, 10 and 13 years, respectively), while the opposite trend was apparent for specificity (0.93, 0.89 and 0.86 for all-cause death versus 0.85, 0.89 and 0.89 for dementia incidence at 5, 10 and 13 years, respectively). The clinically supervised models for dementia incidence had marginally lower PR-AUC (0.17, 0.27 and 0.29 at 5, 10 and 13 years, respectively) compared to the data-driven models. Whilst the PPV was worse (0.13, 0.25 and 0.28 at 5, 10 and 13 years, respectively), the NPV (0.99, 0.97 and 0.96 at 5, 10 and 13 years, respectively) and sensitivity were improved (0.76, 0.62 and 0.55 at 5, 10 and 13 years, respectively).

**Table 2:** Performance table on internal validation sets for prediction of incident dementia and all-cause mortality, after model calibration

Model	ROC-AUC (95% CI)	PR-AUC (95% CI)	PPV (95% CI)	NPV (95% CI)	Sensitivity (95% CI)	Specificity (95% CI)	F1-score threshold (%)
Dementia incidence							
Data-driven							
5-year	0.888 (0.881–0.895)	0.171 (0.130–0.226)	0.140 (0.132–0.148)	0.991 (0.990–0.992)	0.756 (0.732–0.778)	0.854 (0.850–0.857)	18
10-year	0.866 (0.860–0.871)	0.277 (0.235–0.320)	0.260 (0.249–0.271)	0.970 (0.968–0.971)	0.584 (0.566–0.603)	0.889 (0.886–0.892)	20
13-year	0.847 (0.841–0.853)	0.304 (0.264–0.344)	0.292 (0.281–0.304)	0.956 (0.954–0.958)	0.528 (0.512–0.545)	0.890 (0.887–0.893)	20
Clinically supervised							
5-year	0.882 (0.874–0.890)	0.178 (0.138–0.205)	0.134 (0.127–0.142)	0.991 (0.990–0.992)	0.758 (0.735–0.781)	0.847 (0.844–0.850)	18
10-year	0.862 (0.856–0.868)	0.265 (0.224–0.306)	0.247 (0.237–0.257)	0.972 (0.970–0.974)	0.616 (0.598–0.634)	0.873 (0.870–0.877)	19
13-year	0.842 (0.837–0.848)	0.286 (0.247–0.325)	0.279 (0.269–0.290)	0.958 (0.956–0.960)	0.551 (0.534–0.568)	0.877 (0.874–0.880)	19
All-cause mortality							
Data-driven							
5-year	0.891 (0.885–0.896)	0.444 (0.402–0.485)	0.429 (0.414–0.443)	0.949 (0.947–0.952)	0.494 (0.479–0.510)	0.933 (0.930–0.936)	26
10-year	0.885 (0.881–0.889)	0.657 (0.629–0.686)	0.590 (0.580–0.601)	0.910 (0.906–0.913)	0.667 (0.658–0.677)	0.893 (0.889–0.896)	31
13-year	0.882 (0.878–0.886)	0.742 (0.719–0.765)	0.643 (0.634–0.651)	0.884 (0.880–0.888)	0.716 (0.708–0.724)	0.862 (0.859–0.866)	35
Clinically supervised							
5-year	0.849 (0.842–0.855)	0.444 (0.403–0.485)	0.429 (0.414–0.443)	0.945 (0.942––0.947)	0.505 (0.490–0.519)	0.912 (0.909–0.915)	23
10-year	0.857 (0.852–0.861)	0.671 (0.643–0.698)	0.606 (0.596–0.616)	0.900 (0.897–0.903)	0.636 (0.626–0.645)	0.863 (0.859–0.867)	30
13-year	0.860 (0.856–0.864)	0.751 (0.728–0.774)	0.656 (0.648–0.664)	0.872 (0.869–0.876)	0.688 (0.680–0.696)	0.856 (0.852–0.860)	33

Confidence intervals (95% CI) were estimated using the DeLong test without resampling. The probability threshold maximizing the F1-Score (highest potential effectiveness in the context of PR curves) was used to select the best cutoff for estimating PPV, NPV, sensitivity and specificity. ROC-AUC, receiver operating characteristic (ROC) area under-the-curve; PR-AUC, precision-recall (PR) area under-the-curve; PPV, positive predictive value; NPV, negative predictive value.

**Figure 3:**
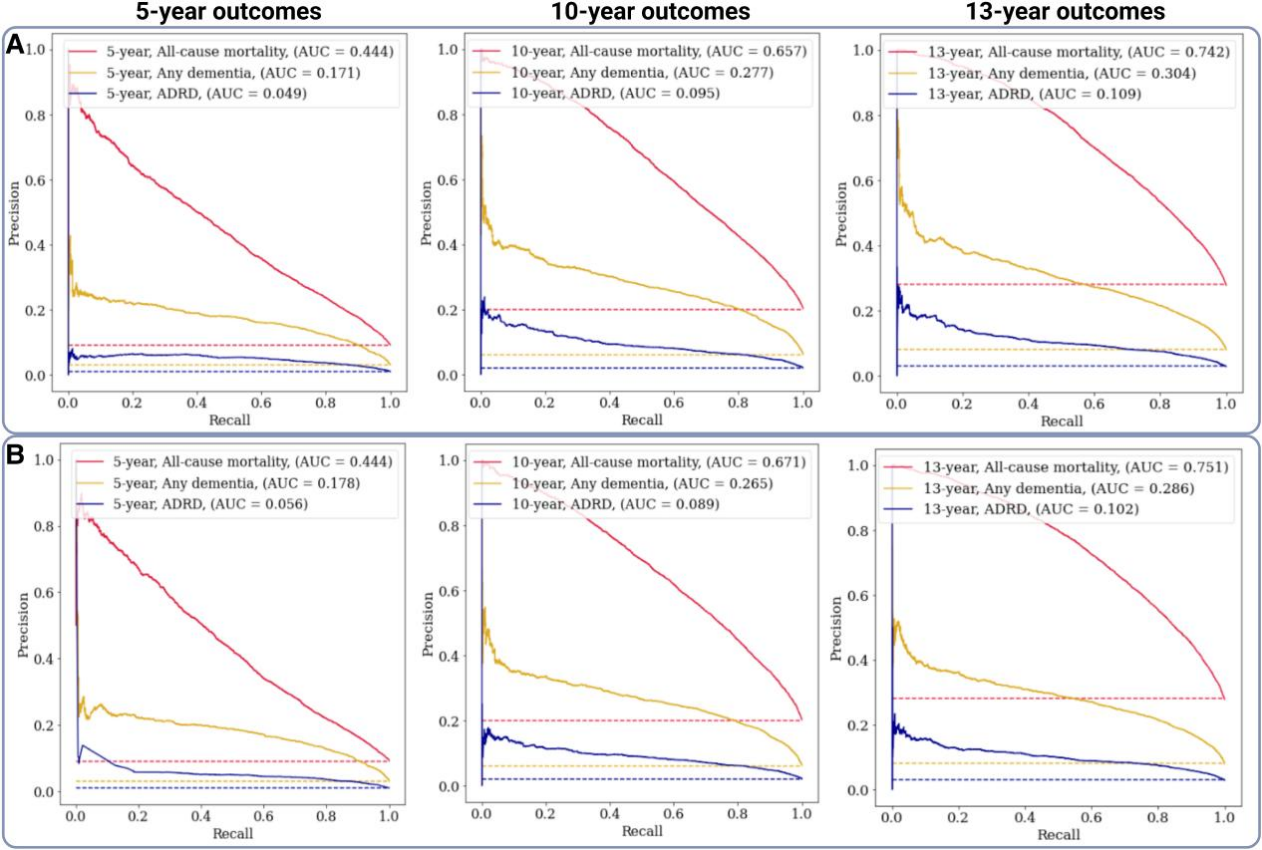
**PR curves showing the calibrated performance [area-under-the-curve (AUC)] in the identification of positive cases in non-specific coded dementia, ADRD and all-cause mortality**. Models in **A** include the data-driven feature subset, while models shown in **B** include the predictive performance on the clinically supervised subset. Horizontal dashed lines show the prevalence of the respective outcome (baseline for random choice), matched by the colour of the curve. Samples in validation set: *n* = 43 234. The overall prevalence of dementia was *n* = 1300 (3%) in any diagnosis, *n* = 416 (1%) in ADRD diagnosis, *n* = 4032 (9%) in all-cause mortality for 5-year outcomes; *n* = 2669 (6%) in any diagnosis, *n* = 936 (2%) in ADRD diagnosis, *n* = 8761 (20%) in all-cause mortality for 10-year outcomes; *n* = 3433 (8%) in any diagnosis, *n* = 1235 (3%) in ADRD diagnosis, *n* = 12 022 (28%) in all-cause death for 13-year outcomes.

There were 4162 ADRD-specific diagnoses within the full prediction window, representing 37% of the total diagnoses of dementia. In this cohort, baseline characteristics were similar, with slight variation in some cardiovascular risk factors (Supplementary Table 4). In the ADRD-specific sensitivity analysis, performance was poorer than for any dementia diagnosis at all prediction windows (Supplementary Table 5). Using the clinically supervised model, the PR-AUCs were 0.05, 0.09 and 0.10 for ADRD compared to 0.17, 0.27 and 0.29 for any dementia at 5, 10 and 13 years, respectively. PR curves comparing performance for any incident dementia, ADRD and all-cause mortality are shown in Fig. 3. ROC curves visualized across the same model sub-types showed similar discrimination (Supplementary Fig. 4). The 10-fold cross-validated ROC and PR curves indicated comparable performance to the internal validation sets for outcomes of future dementia (Supplementary Fig. 5). Model calibration improved precision by correcting for over-estimation of risk in the baseline models, with the effects of the spline calibration in adjusting the output probability scores becoming more pronounced as the prediction windows increased (Supplementary Fig. 6). Model performance over other common supervised ML classifiers is shown in Supplementary Fig. 7, demonstrating optimal *ROC-AUC* and *PR-AUC* using the XGBoost model compared to other approaches tested.

### Stratified analysis in age groups and deprivation status

More diagnoses of dementia were made in individuals from the least deprived SIMD groups, and this relationship was stable over time and by sex (Supplementary Fig. 8). To investigate underlying model bias, the performance of the clinically supervised model for any incident dementia at 13 years was stratified by age and SIMD groups (Table 3). Precision for any dementia diagnosis was notably lower in the smaller number of younger-onset cases (*PR-AUC* of 0.025 [0.015–0.035], *PPV* of 0.047 [0.029–0.075] in those below 60 versus *PR-AUC* of 0.366 [0.332–0.400], *PPV* of 0.320 [0.302–0.332] in those between 80 and 89). The group >90 years old achieved a lower PR-AUC score (0.296 [0.196–0.397]) than the 80- to 89-year-old group, but had a better balance between sensitivity (0.731 [0.623–0.817]) and specificity (0.603 [0.553–0.650]). The PR curves were notably more stable across the individual deprivation quintiles (Supplementary Fig. 9).

**Table 3:** Stratified analysis in 13-year dementia incidence model using clinically supervised features, performed across age groups and level of deprivation (measured in quintiles)

Subset	ROC-AUC (95% CI)	PR-AUC (95% CI)	PPV (95% CI)	NPV (95% CI)	Sensitivity (95% CI)	Specificity (95% CI)	F1-score threshold (%)
Age group							
50–59	0.720 (0.682–0.758)	0.025 (0.015–0.035)	0.047 (0.029–0.075)	0.992 (0.990–0.993)	0.097 (0.061–0.152)	0.982 (0.980–0.984)	3
60–69	0.693 (0.674–0.711)	0.116 (0.062–0.170)	0.124 (0.111–0.139)	0.957 (0.953–0.961)	0.355 (0.321–0.390)	0.851 (0.845–0.858)	9
70–79	0.639 (0.625–0.654)	0.287 (0.267–0.308)	0.256 (0.242–0.270)	0.865 (0.854–0.875)	0.641 (0.616–0.665)	0.553 (0.541––0.565)	19
80–89	0.628 (0.608–0.648)	0.366 (0.332–0.400)	0.320 (0.302–0.332)	0.841 (0.817–0.863)	0.838 (0.813–0.861)	0.325 (0.306–0.343)	23
90+	0.728 (0.675–0.781)	0.296 (0.196–0.397)	0.269 (0.214–0.332)	0.918 (0.878–0.946)	0.731 (0.623–0.817)	0.603 (0.553–0.650)	20
SIMD (Quintiles): 1—most deprived, 5—least deprived							
Group 1	0.801 (0.781–0.821)	0.260 (0.192–0.329)	0.272 (0.241–0.304)	0.945 (0.936–0.952)	0.553 (0.502–0.602)	0.838 (0.825–0.850)	19
Group 2	0.816 (0.802–0.831)	0.313 (0.264–0.363)	0.301 (0.278–0.326)	0.945 (0.939–0.951)	0.596 (0.560–0.631)	0.834 (0.824–0.843)	20
Group 3	0.810 (0.792–0.829)	0.306 (0.243–0.370)	0.284 (0.257–0.313)	0.947 (0.940–0.953)	0.562 (0.518–0.605)	0.846 (0.836–0.857)	19
Group 4	0.833 (0.818–0.848)	0.302 (0.251–0.353)	0.279 (0.254–0.306)	0.954 (0.947–0.959)	0.574 (0.532–0.614)	0.855 (0.846–0.864)	19
Group 5	0.834 (0.822–0.845)	0.265 (0.224–0.307)	0.256 (0.238–0.275)	0.958 (0.954–0.962)	0.577 (0.546–0.609)	0.852 (0.845–0.858)	18

Confidence intervals (95% CI) were estimated using the DeLong test without resampling. The probability threshold maximizing the F1-Score (highest potential effectiveness in the context of precision-recall curves) was used to select the best cutoff for estimating PPV, NPV, sensitivity and specificity. ROC-AUC, receiver operating characteristic (ROC) area under-the-curve; PR-AUC, precision-recall (PR) area under-the-curve; PPV, positive predictive value; NPV, negative predictive value; SIMD, Scottish Index for Multiple Deprivation.

### Risk factors and prediction ranking

**Figure 4:**
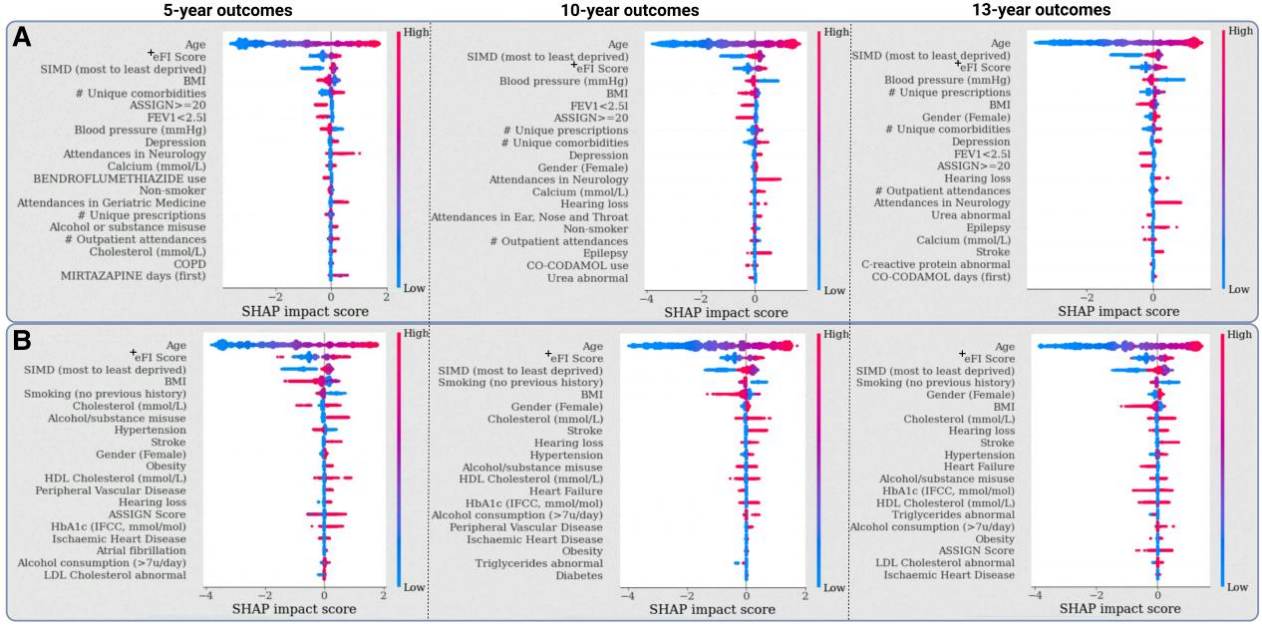
**SHAP feature importance summaries in all dementia model instances.** Density plots include the top 20 ranked predictors (from best to worst, independent of importance direction). Cumulative Shapley values were generated by the TreeSHAP algorithm and computed on the patient level. (**A**) Importance plot on the data-driven feature subset. (**B**) Importance plot on the clinically supervised feature subset. Samples in validation set: *n* = 43 234. eFI, modified electronic Frailty Index; SIMD, Scottish Index for Multiple Deprivation; COPD, Chronic Obstructive Pulmonary Disease; ASSIGN, cardiovascular risk calculator validated in Scottish populations (Scottish Heart Health Extended Cohort); FEV1, diagnostic measure of forced expiratory volume in 1 s (values below 2.5 L may suggest reduced expiratory airflow); IFCC, International Federation of Clinical Chemistry. ^+^Excludes deficits coded as ‘Memory & Cognitive problems’ from the estimation.

**Figure 5:**
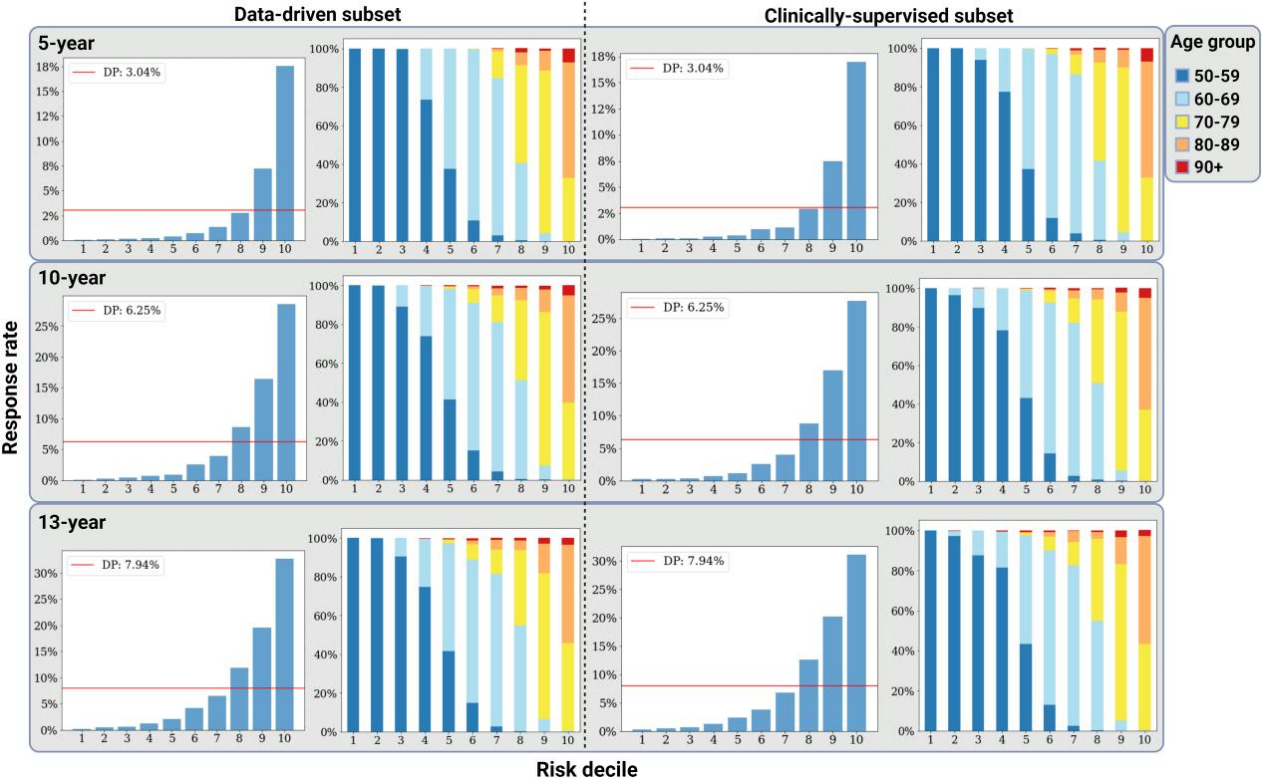
**Prediction decile analysis detailing the response rate (captured individuals with future diagnosis) in each dementia incidence model after risk stratification of the output probabilities into 10 equally sized groups**. The age-stratified plots indicate the age distribution per risk decile over the internal validation set. The horizontal straight line refers to the overall prevalence of the diagnosis in each model subset and respective validation set. Overall sample size: *n* = 43 234 (*n* = 18 352 in 50–59 group; *n* = 13 170 in 60–69 group; *n* = 7867 in 70–79 group; *n* = 3377 in 80–89 group; *n* = 468 in 90+ group). DP, diagnosis prevalence in validation set.

We used the SHAP framework to examine the top 20 ranked predictors of future dementia diagnosis in both the clinically supervised and data-driven models (Fig. 4). In all instances, age (older), deprivation status (least deprived) and eFI (higher frailty) were among the top features associated with increased risk of dementia. Within the data-driven models, a range of variables were linked to a higher likelihood of a future diagnosis, including a higher number of long-term conditions, number of prescriptions, depression, hearing loss, epilepsy, stroke, smoking history, elevated calcium, glucose and cholesterol, and clinic reviews within neurology and geriatric medicine services. On the other hand, a documented increased cardiovascular risk (ASSIGN score ≥20), poorer lung function, higher blood pressure, high BMI, use of anti-hypertensive drugs and abnormal urea blood results appeared protective. Within the clinically supervised subsets, the variation in SHAP scores was more pronounced. Prior hearing loss, alcohol or substance misuse, stroke, obesity, peripheral vascular disease as well as smoking were associated with increased risk of future diagnosis. A history of hypertension appeared protective in 5-year outcomes but was associated with increased risk of dementia incidence over longer periods. On the other hand, heart failure was protective over 10 and 13 years, but this may be biased by survival rates in these patients. After risk stratification, both clinically supervised and data-driven models had predictions that reached an incidence rate above 30% at 13 years in the highest risk decile, compared to a whole-population incidence of 8% (Fig. 5). Over 40% of this group were in their 70s at the time of index risk prediction. Conversely, 13-year dementia risk was <1% in the lowest three prediction deciles, although these predominantly consisted of individuals in their 50s at the time of risk prediction.

## Discussion

We have extensively evaluated the diagnostic quality of a machine learning prediction model for long-term dementia risk developed from entirely routinely collected data. We demonstrate moderately capable prediction for diagnoses up to 13 years later, which could inform further testing or risk factor surveillance in those at the extreme of predicted risk. There was marginally improved PR with a high variable count data-driven approach using XGBoost, but at the expense of rule-out performance compared to a clinically supervised model restricted to 22 variables. Precision was consistent across quintiles of socioeconomic deprivation, but detection of younger-onset dementia cases earlier than 70 years old was notably limited, and performance fell markedly when restricted to more specific ADRD-coded diagnoses. Early detection of dementia is a major societal challenge, but population-level screening using routinely collected data to identify high-risk subgroups may improve the targeting of resource-intensive dementia investigations.

Our study has important strengths. We used a large, population-level dataset with integrated primary and secondary care health data to maximize ascertainment of risk factors and dementia diagnoses over 13 years of follow-up. We were conservative in identifying a model development cohort at low risk of established cognitive issues by exclusion criteria using hundreds of codes and clinic attendances suitable for identifying pre-diagnostic dementia. In contrast to many reports of machine learning models for risk prediction, we have presented performance beyond basic discrimination and calibration measures flattered by relatively low outcome incidence, using PR to demonstrate the clear challenge of confident prediction for this complex condition. We have also shown a direct comparison between a data-driven approach and clinically supervised selection, suggesting in this case that the latter provides similar performance with parsimony and, therefore, greater potential for transferability to other EHR systems.

The challenge of managing dementia-related disorders across an ageing population cannot be overstated. The anticipated increase in the number of individuals living with dementia by 2050 is likely to be in the region of 166%.^31^ EHR data have led to a surge of studies covering large integrated population-level data to understand disparity, adverse effects and outcomes in those affected by dementia and related conditions.^32-34^ These data contain important long-term markers of health to understand disease progression. Highlighting the cumulative effects of these markers and ranking their contribution to a potential diagnosis can be used to improve the prioritization of public health measures in middle-aged populations. However, this knowledge often lacks understanding of individualized risk, which is essential in an era of precision medicine to drive shared treatment decisions between an individual and their clinician. The great potential of data-driven prediction is coming closer to realization with larger EHR datasets that are crucially more granular in detail to understand heterogeneity of risk, using ever-advancing machine learning methods.

The predictive quality and precision of our models for dementia improved as the prediction windows lengthened, highlighting the long-term cumulative effect of many risk factors associated with dementia pathology. Inevitably in an observational study, coding of disease or lifestyle factors reflects engagement with health services and is at risk of ascertainment bias. Despite this, our final model achieved sufficient precision-recall in the highest decile of risk to identify individuals with a 1 in 3 risk of a dementia diagnosis within 13 years, compared to baseline population risk of 1 in 13. While it might be expected that many of these individuals would be of advanced age, over 40% were under 80 years old. So, although the PPV was generally limited, in the context of a general population, high-risk stratification could still provide substantial public benefits. These may include better health and care resource utilization and improved targeting of pharmacological treatments for primary prevention.^35,36^ The length of the prediction window is relevant for modern Alzheimer’s dementia immunotherapy, where confirmation of amyloid deposition and treatment are needed many years prior to established cognitive symptoms.^6,7^ Population screening using predictive models of future risk might offer a more equitable strategy for determining eligibility for PET-imaging or novel therapies, in contrast to the potential for access favouring those with financial means or sufficient healthcare literacy.

Here, we must acknowledge some critical challenges with the use of routinely collected data in model development. We have shown higher rates of dementia diagnoses in those with the least socioeconomic deprivation, which is in sharp contrast to selected cohort studies such as those in UK Biobank, the Whitehall II study of UK civil servants and Finnish cohorts.^37,38^ Our data are likely to reflect stronger health-seeking behaviours for early cognitive decline in more advantaged populations, but the competing risk of earlier death in those from more disadvantaged backgrounds must also be considered. Further, the stratified analysis of our prediction model showed stable predictive performance across deprivation groups, suggesting potential utility even if under-representative in higher deprivation groups. Interestingly, the added value of SIMD measures is well recognized in the prediction of long-term cardiovascular events using the ASSIGN score.^25^ Ultimately, our data reflect the true known population burden of dementia within a National Health Service in the UK where access to testing or diagnosis is not limited by ability to pay or requirement for insurance. Cohort studies have their own issues with representative inclusion, so caution must be taken against over-interpretation in this area. The lower likelihood of obtaining a dementia diagnosis in people from poorer backgrounds is a challenge for all healthcare systems but contributes to the argument for population screening and proactive targeting if and when more effective treatments for earlier dementia are available. Even without novel immunotherapies there are likely to be other societal advantages to system-wide recognition of early cognitive decline, to maximize access to appropriate health and social care service support and benefits where needed.

While various clinical models have been developed in the past, the evidence suggests that there is no single best prognostic model for dementia prediction. Only a small proportion of such models have been externally validated. One example is the Cardiovascular Risk Factors, Ageing and Dementia model, which showed low discrimination power for prediction of incident dementia, with a ROC-AUC of 0.71 (95% CI 0.66–0.76).^39,40^ Additionally, models that include cognitive testing as a predictor tend to have higher ROC-AUC scores (>0.75) compared to those that do not.^41^ However, this renders them unsuitable for pre-symptomatic screening. This limitation is also present in most ML studies. Some EHR-based studies have demonstrated exceptional performance with ROC scores of 0.89 and above for long-term predictions, but target only patients with memory clinical referrals.^17^ Other existing data studies achieved ROC-AUCs over 0.80 at 6 years prior to diagnosis using propensity-matched cohorts.^42^ However, this approach may limit generalizability, as it typically discards many control cases that could contain important risk indicators. Through our approach, we opted for evaluating an unbiased sample of community-dwelling adults using the PR-AUC, which effectively measures discrimination when sample sizes are imbalanced. Thus, the variability in study design settings and reported outcomes makes it difficult to establish a clinical or data-driven performance baseline for comparison against our models.

The estimated SHAP values indicated a wide range of routine data points associated with dementia risk. While a lot of model assumptions regarding modifiable risk factors were consistent with reports from the literature^9^ (e.g. smoking, alcohol consumption, hearing loss, stroke and epilepsy), there were also some contradictions (e.g. heart failure being protective of long-term diagnoses and hypertension being protective of short-term incidence). Although these may be partially explained by underreporting in EHR data or competing risks of death from acute conditions, there may also be a causal effect stemming from better control measures provided for high-risk individuals.^43,44^ Nonetheless, frailty and lifestyle risk factors (age, eFI, SIMD, blood pressure, smoking and BMI) unsurprisingly headlined the summary plots. In these cases, frailty in dementia was associated with a high number of ageing-related health deficits and signs of lower BMI and blood pressure.

We also acknowledge several limitations of our study. Firstly, our resources did not allow patient and public engagement to be incorporated into the study design. Although we have been robust in cross-validation, our models lack external dataset validation. However, our clinically supervised model of 22 variables has a high transferability potential to achieve this. Due to the underreporting of PR-AUC in dementia studies, and lack of established prediction baselines, we could not compare performance against similar studies in the literature. We utilized phenotype code lists to derive confirmed clinical diagnoses of dementia from GP and hospital coding. Although the accuracy of EHR-based phenotype definitions is typically high,^45^ there is also a possibility that these are underreported across the general population. Furthermore, our analysis highlighted difficulties in developing models for specific ADRD diagnoses that were clearly under-reported in our data. ADRD represents the most common dementia sub-type, but only around a third of dementia cases in our cohort reflected this, with a high proportion of unspecified dementia codes used particularly in primary care. Our relatively low numbers of younger-onset dementia diagnoses limited the validity of prediction in this group. This was partially due to the data collection procedure, as we defined a fixed cutoff of individuals aged 50 and above on 1st April 2009, with no additional entries after this date. This further prevented training and validation on shorter follow-ups beyond the first study year, as it would limit applicability for younger individuals. Future models could incorporate competing risk components to better account for the potential overestimation of dementia risk in our models for people at simultaneously higher risk of earlier non-dementia-related death. This approach has been integrated into newer cardiovascular risk models such as SCORE2.^46^

## Conclusion

Strategies to improve primary prevention for dementia are essential to mitigate the challenges of an ageing population. We have demonstrated that gradient-boosting (XGBoost) machine learning prediction models, based entirely on routinely collected health data, can provide moderately capable prediction for high-risk individuals many years prior to dementia diagnosis, including when using a parsimonious clinically supervised model with high transferability. Personalized estimates of future dementia risk could influence risk factor modification, access to clinical trials and help target brain imaging required for novel immunotherapy treatments in selected individuals with pre-symptomatic disease.

## Supplementary Material

fcae469_Supplementary_Data

## Data Availability

The data that support the findings of this study are not openly available due to reasons of sensitivity, but summary data and visual diagrams can be provided by the corresponding author upon reasonable request. Data and models are located in a Secure Data Environment provided by the DataLoch service. The code used to generate the findings in this study is not publicly available due to proprietary restrictions applicable to industry partners Red Star. However, specific elements of the analysis code may be made available upon reasonable request to the corresponding author, subject to approval by RedStar and confidentiality agreements.
