# Supplementary material for: Predicting incident dementia in community-dwelling older adults using primary and secondary care data from electronic health records
Source: Brain Commun. 2024 Dec 24;7(1):fcae469. doi: 10.1093/braincomms/fcae469 (PMC11697165; doi:10.1093/braincomms/fcae469)
Supplement: fcae469_Supplementary_Data [file fcae469_supplementary_data.pdf]

SUPPLEMENTARY MATERIALS

Contents:

Supplementary Table 1. Feature definitions, classifications and criteria for collection for prediction of dementia incidence relative to Index Prediction Date (IPD).
Supplementary Table 2. List of fine-tuned model hyperparameters for the dementia incidence models (tested on the data-driven subset).
Supplementary Table 3. Training and validation set characteristics after performing a random stratified split on age, mortality and dementia prevalence.
Supplementary Table 4. Patient characteristics at baseline grouped by presence of ADRD-coded diagnosis.
Supplementary Table 5. Performance table on internal validation sets for prediction of ADRD (Alzheimer's disease-related dementia) incidence, after model calibration.
Supplementary Fig. 1. Annual distribution of missingness across some of the clinically-curated data variables for predicting dementia incidence.
Supplementary Fig. 2. The annual distribution of coded dementia diagnoses in unique patients from the processed NHS Lothian cohort.
Supplementary Fig. 3. Forest plot showing the log-transformed hazard ratio (95% CI) estimates in a multivariate Cox regression model for incidence of dementia using the clinically-supervised feature set (excluding age).
Supplementary Fig. 4. Receiver Operating Characteristic (ROC) curves showing the calibrated positive discrimination performance (Area under-the-curve [AUC]) in non-specific coded dementia, ADRD and all-cause mortality.
Supplementary Fig. 5. Stratified 10-fold cross-validation results for predicting any incident dementia across each target window.
Supplementary Fig. 6. Calibration curves detailing the relationships between the model probability scores and the actual event rate of dementia, as well as the absolute probability distributions before and after the calibration.
Supplementary Fig. 7. Performance comparison across the Receiver Operating Characteristic (ROC) and Precision-Recall area-under-the-curves in various commonly adapted Machine Learning classifiers.
Supplementary Fig. 8. Exploratory data analysis detailing inequalities by deprivation status in patients with dementia incidence.
Supplementary Fig. 9. Stratified analysis results comparing the Precision-Recall curves across age and socioeconomic deprivation groups on the clinically-supervised feature subset.

Supplementary Table 1. Feature definitions, classifications and criteria for collection for prediction of dementia incidence relative to Index Prediction Date (IPD). In all dementia models the IPD is defined as: 1st Apr 2010. SIMD – Scottish Index for Multiple Deprivation.

Group	Data types	Temporal (sampling) criteria	Variable thresholds	In curated set (Y/N)
**Demographics**				
**Age**	Continuous	At IPD	/	Y
**Sex**	Categorical	At IPD	1 [Female],<br>0 [Male]	Y
**SIMD (Quntiles)**	Ordinal	At IPD	/	Y
**Modifiable lifestyle factors**				
**Smoking**	Categorical	Last report prior to IPD	Ex-smoker,<br>Non-smoker,<br>Current smoker	Y
**Alcohol consumption**	Categorical	Last report prior to IPD	Harmful levels [>7u/day]	Y
**Body Mass Index**	Continuous (Last measure) and Categorical	Last measure prior to IPD	>25 [overweight] and >30 [obesity]	Y
**Lab tests**				
eGFR (ml/min/1.73m^2),<br>Potassium (mEq/L),<br>Creatinine (mg/dL),<br>Urea (mg/dL),<br>Sodium (mEq/L),<br>Calcium (mmol/L),<br>TCO2 (pH),<br>Chloride (mmol/L),<br>Lymphocyte count (x10^9/L),<br>MCH (pg/cell),<br>Basophil count (x10^9/L),<br>Haematocrit (L/L),<br>Monocyte count (x10^9/L),<br>Eosinophil count (x10^9/L),<br>Neutrophil count (x10^9/L),<br>Red cell count (x10^9/L),	Continuous (test value),<br>Categorical (normal/abnormal),<br>Temporal (days since last test from IPD)	Last recorded test prior to IPD	-1 [below reference range],<br>0 [normal],<br>1 [above reference range]	N<br>N<br>N<br>N<br>N<br>N<br>N<br>N<br>N<br>N<br>N<br>N<br>N<br>N<br>N<br>N

MCV (µm3),<br>Platelet count (x10^9/L),<br>White cell count (x10^9/L),<br>Haemoglobin (g/dl),<br>MCHC (g/dl),<br>C-reactive protein (mg/L),<br>Lactate (mmol/L),<br>ESR (mm/hr),<br>Ferritin (µg/L),<br>Total cholesterol (mmol/L),<br>HDL cholesterol (mmol/L),<br>LDL cholesterol (mmol/L),<br>Triglycerides (mmol/L),<br>Bilirubin (µmol/L),<br>Albumin (g/dL),<br>Alkaline Phoshpatase (U/L),<br>ALT (IU/L),<br>GGT (IU/L),<br>AST (IU/L),<br>Glucose (mmol/L),<br>HbA1c (IFCC, mmol/mol)				N<br>N<br>N<br>N<br>N<br>N<br>N<br>N<br>N<br>Y<br>Y<br>Y<br>Y<br>N<br>N<br>N<br>N<br>N<br>N<br>N<br>Y
**Prescriptions**				
Alendronic Acid, Allopurinol, Amitriptyline, Amlodipine, Aspirin, Atenolol, Atorvastatin, Bendroflumethiazide, Bisoprolol Fumarate, Blood Glucose Testing Strips, Candesartan Cilexetil, Citalopram, Clopidogrel, Co-codamol, Co-dydramol, Diazepam, Diclofenac, Digoxin, Dihydrocodeine Tartrate, Doxazosin, Enalapril Maleate, Ferrous Fumarate, Finasteride, Fluoxetine, Folic Acid, Furosemide, Gabapentin, Gliclazide, Glyceril Trinitrate, Ibuprofen, Isosorbide Mononitrate, Lisinopril, Losartan Potasssium, Metformin Hydrochloride, Mirtazapine, Morphine, Nifedipine, Paracetamol, Prednisolone, Pregabalin, Ramipril, Rosuvastatin, Sertraline, Simvastatin, Solifenacin, Tamsulosin Hydrochloride, Tramadol Hydrochloride, Warfarin Sodium, Zopiclone	Continuous (# prescribed total),<br>Continuous (# prescribed by drug),<br>Temporal (Days since first prescribed by drug from IPD),<br>Temporal (Days since last prescribed by drug from IPD)	prior to IPD	/	N
**Outpatient attendances (by specialty)**				
General Medicine, Cardiology, Endocrinology, Diabetes, Gastroenterology, Geriatric Medicine, Medical Oncology, Renal Medicine, Neurology, Palliative Medicine, Rehabilitation Medicine, Respiratory Medicine, Rheumatology, General Surgery, Non-vascular	Continuous (# total attendances),<br>Continuous (# attendances by specialty),	prior to IPD	/	N

Surgery, Vascular Surgery, Anaesthetics, Cardiac Surgery, Thoracic Surgery, Ear/nose/throat, Neurosurgery, Opthalmology, Trauma and Orthopaedic Surgery, Urology, Oral Surgery, Oral Medicine, Clinical Oncology, Haematology	Continuous (# failed attendances by specialty)			
**Comorbidity history (SMR/GP READ coded)**				
Lung cancer,<br>Stable angina,<br>Multple myeloma,<br>Transient Ischaemic attack,<br>Tinnitus,<br>Schizophrenia,<br>Diabetic opthalmic complications,<br>Osteoarthritis,<br>Hearing loss,<br>Liver cancer,<br>Prostate cancer,<br>Parkinson's disease,<br>Heart failure,<br>Leukaemia,<br>Breast cancer,<br>Retroperitoneal cancer,<br>Visual impairment/blindness,<br>Liver fibrosis/sclerosis/cirrhosis,<br>Bone cancer,<br>Spinal stenosis,<br>COPD,<br>Rheumatoid Arthritis,<br>Peripheral Vascular Disease,<br>Ischaemic Heart Disease,<br>Ovarian cancer,<br>Osteoporosis,<br>Lymph nodes cancer,<br>End-stage Renal disease,<br>Asthma,<br>Atrial Fibrillation,<br>Bronchiestasis,<br>Epilepsy,<br>Non-hodgkin lymphoma,<br>Myocardial infarction,<br>Hip fracture,	Continuous (Total # conditions), Categorical (Presence/absence of diagnosis)	Any diagnosis prior to IPD	1 [any previous GP or SMR-coded diagnosis], 0 [no prior diagnosis]	N<br>N<br>N<br>N<br>N<br>N<br>N<br>N<br>Y<br>N<br>N<br>N<br>Y<br>N<br>N<br>N<br>N<br>N<br>N<br>N<br>N<br>N<br>Y<br>Y<br>N<br>N<br>N<br>N<br>N<br>Y<br>N<br>N<br>N<br>N<br>N

Psychoactive substance misuse,<br>Hypertension,<br>Depression,<br>Obesity,<br>Lung and trachea cancer,<br>Diabetes,<br>Other organ cancer,<br>Alcohol/substance misuse,<br>Alcoholic liver disease,<br>Bipolar affective disorder,<br>Colorectal/anus cancer,<br>Stroke				N<br>Y<br>N<br>Y<br>N<br>Y<br>N<br>Y<br>N<br>N<br>N<br>Y
**Other routine measurements**				
Blood pressure (mmHg)	Continuous (Last measure), Categorical (high measures)	Last measure prior to IPD	1 if >140/90 mmHg, 0 otherwise	N
Electronic Frailty Index (eFI)	Continuous, Categorical (moderate high, high frailty)	Last measure prior to IPD	Moderate high: eFI>0.23, High: eFI>0.35	Y
ASSIGN cardiovascular risk score	Continuous (Last measure), Categorical (at 10-year risk of cardiovascular disease)	Last measure prior to IPD	At 10-year risk: ASSIGN >=20	Y
Pulmonary function: Forced expired volume in 1 second (FEV1 [l] and % predicted [ppFEV1])	Continuous, Categorical (moderate or severe airflow obstruction)	Last measure prior to IPD	1 if FEV1 < 2.5l or ppFEV1 < 50%, 0 otherwise	N

Supplementary Table 2. List of fine-tuned model hyperparameters for the dementia incidence models (tested on the data-driven subset). 'aucpr' – Precision-Recall Area-under-the-curve, the optimisation target for the loss function; 'scale_pos_weight' is a constant scaling parameter applied to each training record with future dementia incidence, estimated as the ratio: $\frac{\#\, participants\ dementia\ free}{\#\, participants\ with\ dementia}$ across each respective training set.

XGBoost model hyperparameter	5-year	10-year	13-year
**Training rounds**	20,000	20,000	20,000
**Early stopping rounds**	100	100	100
**Objective function**	binary:logistic	binary:logistic	binary:logistic
**Evaluation metric**	aucpr	aucpr	aucpr
**Max tree depth (max_depth)**	3	3	3
**Learning rate (eta)**	0.01	0.01	0.01
**Positive class weight (scale_pos_weight)**	32.4	15.3	11.6

Supplementary Table 3. Training and validation set characteristics after performing a random stratified split on age, mortality and dementia prevalence. Values are in proportion of patients (%) unless stated otherwise. Unadjusted P-values for continuous variables were generated using the Kruskal-Wallis H test, while the chi-squared test was used for categorical variables. SIMD – Scottish Index for Multiple Deprivation. ADRD – Alzheimer's disease-related dementia diagnosis, as coded in phenotypes data.

Characteristic	Missing	All (n=144,113)	Training (n=100,879)	Validation (n=43,234)	p
**Age (median, IQR)**	0	61 (55, 70)	61 (55, 70)	61 (55, 70)	0.831
**Age group**	0	..	..	..	1.000
**50-59**	..	61,771 (42%)	42,819 (42%)	18,352 (42%)	..
**60-69**	..	43,903 (31%)	30,733 (31%)	13,170 (31%)	..
**70-79**	..	26,219 (18%)	18,352 (18%)	7,867 (18%)	..
**80-89**	..	11,258 (8%)	7,881 (8%)	3,377 (8%)	..
**90+**	..	1,562 (1%)	1,094 (1%)	468 (1%)	..
**Sex**	0	..	..	..	0.664
**Male**	..	68,839 (48%)	48,149 (48%)	20,690 (48%)	..
**Female**	..	75,274 (52%)	52,730 (52%)	22,544 (52%)	..
**SIMD in quintiles**	32,843	..	23,118 (23%)	9,725 (23%)	0.072
**1 (most deprived)**	..	13,194 (9%)	9,345 (9%)	3,849 (9%)	..
**2**	..	22,070 (15%)	15,376 (15%)	6,694 (16%)	..
**3**	..	16,932 (12%)	11,820 (12%)	5,112 (12%)	..
**4**	..	20,272 (14%)	14,104 (14%)	6,168 (14%)	..
**5 (least deprived)**	..	38,802 (27%)	27,116 (27%)	11,686 (27%)	..
**All-cause mortality**	0	..	..	..	1.000
**5-year**	..	13,437 (9%)	9,405 (9%)	4,032 (9%)	..
**10-year**	..	29,204 (20%)	20,443 (20%)	8,761 (20%)	..
**13-year**	..	40,074 (28%)	28,052 (28%)	12,022 (28%)	..
**Any dementia incidence**	0	..	..	..	1.000
**5-year**	..	4,334 (3%)	3,034 (3%)	1,300 (3%)	..
**10-year**	..	8,899 (6%)	6,230 (6%)	2,669 (6%)	..
**13-year**	..	11,443 (8%)	8,010 (8%)	3,433 (8%)	..
**ADRD incidence**	0	..	..	..	1.000
**5-year**	..	1,384 (1%)	968 (1%)	416 (1%)	0.986
**10-year**	..	3,056 (2%)	2,120 (2%)	936 (2%)	0.456
**13-year**	..	4,162 (3%)	2,927 (3%)	1,235 (3%)	0.653

Supplementary Table 4. Patient characteristics at baseline grouped by presence of ADRD-coded diagnosis. Values are in proportion of patients (%) unless stated otherwise. Unadjusted P-values for continuous variables were generated using the Kruskal-Wallis H test, while the chi-squared test was used for categorical variables. ADRD – Alzheimer's disease-related dementia, SIMD – Scottish Index for Multiple Deprivation, ASSIGN - cardiovascular risk calculator validated in Scottish populations (Scottish Heart Health Extended Cohort), eFI (electronic Frailty Index) – cumulative deficit measure relative to frailty.

Characteristic	Missing	All (n=144,113)	No ADRD diagnosis (n=139,351)	ADRD diagnosis (n=4,162)	p
**Age (median, IQR)**	0	61 (55, 70)	61 (55, 70)	74 (68, 79)	<0.001
**Sex**	0	..	..	..	<0.001
**Male**	..	68,839 (48%)	67,381 (48%)	1,458 (35%)	..
**Female**	..	75,274 (52%)	72,570 (52%)	2,704 (65%)	..
**SIMD in quintiles**	32,843	..	32,422 (23%)	421 (10%)	<0.001
**1 (most deprived)**	..	13,194 (9%)	12,769 (9%)	425 (10%)	..
**2**	..	22,070 (15%)	21,253 (15%)	817 (20%)	..
**3**	..	16,932 (12%)	16,349 (12%)	583 (14%)	..
**4**	..	20,272 (14%)	19,536 (14%)	736 (18%)	..
**5 (least deprived)**	..	38,802 (27%)	37,622 (27%)	1,180 (28%)	..
**Lifestyle and medical risk factors**	..	..	..	..	..
**Smoking (current)**	22,799	26,303 (18%)	25,846 (19%)	457 (11%)	<0.001
**Alcohol (high consumption, >6u/day)**	51,170	828 (0.6%)	820 (0.6%)	8 (0.2%)	<0.001
**BMI (median, IQR)**	40,353	26.60 (23.80, 30.30)	26.67 (23.80, 30.30)	26.40 (23.70, 29.60)	<0.001
**ASSIGN Score (median, IQR)**	140,068	13 [7,21]	13 [7, 21]	21 [14, 30]	<0.001
**Modified eFI Score (median, IQR)***	42,689	0.06 [0.03, 0.11]	0.06 [0.03, 0.11]	0.09 [0.03, 0.14]	<0.001
**Medical condition history**	0	..	..	..	..
**Atrial fibrillation**	..	6,447 (5%)	6,194 (4%)	253 (6%)	<0.001
**Ischaemic Heart Disease**	..	13,655 (10%)	13,025 (9%)	630 (15%)	<0.001
**Heart Failure**	..	4,305 (3%)	4,158 (3%)	147 (4%)	0.041
**Hypertension**	..	48,117 (33%)	46,082 (33%)	2,035 (49%)	<0.001
**Stroke**	..	5,464 (4%)	5,305 (4%)	159 (4%)	0.954
**Peripheral Vascular Disease**	..	3,692 (3%)	3,572 (3%)	120 (3%)	0.200
**Diabetes**	..	12,278 (9%)	11,858 (8%)	420 (10%)	<0.001
**Obesity**	..	18,266 (13%)	17,742 (13%)	524 (13%)	0.886
**Alcohol/substance misuse**	..	8,176 (6%)	8,047 (6%)	129 (3%)	<0.001
**Hearing loss**	..	10,293 (7%)	9,742 (7%)	551 (13%)	<0.001
**Lab tests (median, IQR)**	..	..	..	..	..
**Total cholesterol (mmol/L)**	118,301	4.6 (3.9, 5.5)	4.6 (3.9, 5.5)	4.6 (4.0, 5.4)	0.909
**Triglycerides (mmol/L)**	126,196	1.6 (1.1, 2.3)	1.6 (1.2, 2.3)	1.5 (1.1, 2.0)	<0.001
**HbA1c (mmol/mol)**	138,004	53 (44, 64)	53 (44, 65)	52 (44, 60)	0.279
**Cholesterol ratio (Total / HDL, mg/dL)**	127,734	3.5 (2.9, 4.3)	3.5 (2.9, 4.4)	3.3 (2.8, 4.0)	<0.001
**All-cause mortality**	0	40,074 (28%)	37,627 (27%)	2,447 (59%)	<0.001

* Excludes deficits coded as 'Memory & Cognitive problems' from the estimation.

Supplementary Table 5. Performance table on internal validation sets for prediction of ADRD (Alzheimer's disease-related dementia) incidence, after model calibration. 95% CI were estimated using the DeLong test without resampling. Youden's Index (J-statistic) represents the probability threshold with the highest potential effectiveness based on the Receiver Operating Characteristic (ROC) curve. The threshold maximising the F1-Score (highest potential effectiveness in the context of Precision-Recall curves) was used to select the best cutoff for estimating PPV, NPV and Sensitivity. ROC-AUC – Area under-the-curve of the ROC curve; PR-AUC – Area under-the-curve of the Precision-Recall curve; PPV – Positive Predictive Value; NPV – Negative Predictive Value.

Model	ROC-AUC (95% CI)	PR-AUC (95% CI)	PPV (95% CI)	NPV (95% CI)	Sensitivity (95% CI)	Specificity (95% CI)	F1-Score threshold
**ADRD incidence**							
**Data-driven**							
**5-year**	0.869 (0.843, 0.895)	0.049 (0.031, 0.067)	0.051 (0.044, 0.059)	0.994 (0.993, 0.994)	0.395 (0.350, 0.442)	0.927 (0.924, 0.929)	5%
**10-year**	0.843 (0.828, 0.858)	0.095 (0.030, 0.159)	0.101 (0.091, 0.112)	0.985 (0.984, 0.987)	0.362 (0.331, 0.394)	0.931 (0.929, 0.933)	8%
**13-year**	0.820 (0.810, 0.830)	0.109 (0.067, 0.150)	0.113 (0.104, 0.123)	0.981 (0.979, 0.982)	0.389 (0.362, 0.416)	0.910 (0.908, 0.913)	9%
**Clinically-supervised**							
**5-year**	0.864 (0.850, 0.878)	0.056 (0.036, 0.075)	0.063 (0.052, 0.077)	0.992 (0.991, 0.993)	0.208 (0.172, 0.249)	0.969 (0.968, 0.971)	6%
**10-year**	0.838 (0.827, 0.849)	0.089 (0.040, 0.139)	0.099 (0.088, 0.110)	0.985 (0.984, 0.986)	0.330 (0.300, 0.361)	0.935 (0.933, 0.937)	8%
**13-year**	0.816 (0.806, 0.826)	0.102 (0.046, 0.158)	0.112 (0.103, 0.122)	0.980 (0.979, 0.982)	0.377 (0.351, 0.405)	0.912 (0.910, 0.915)	9%

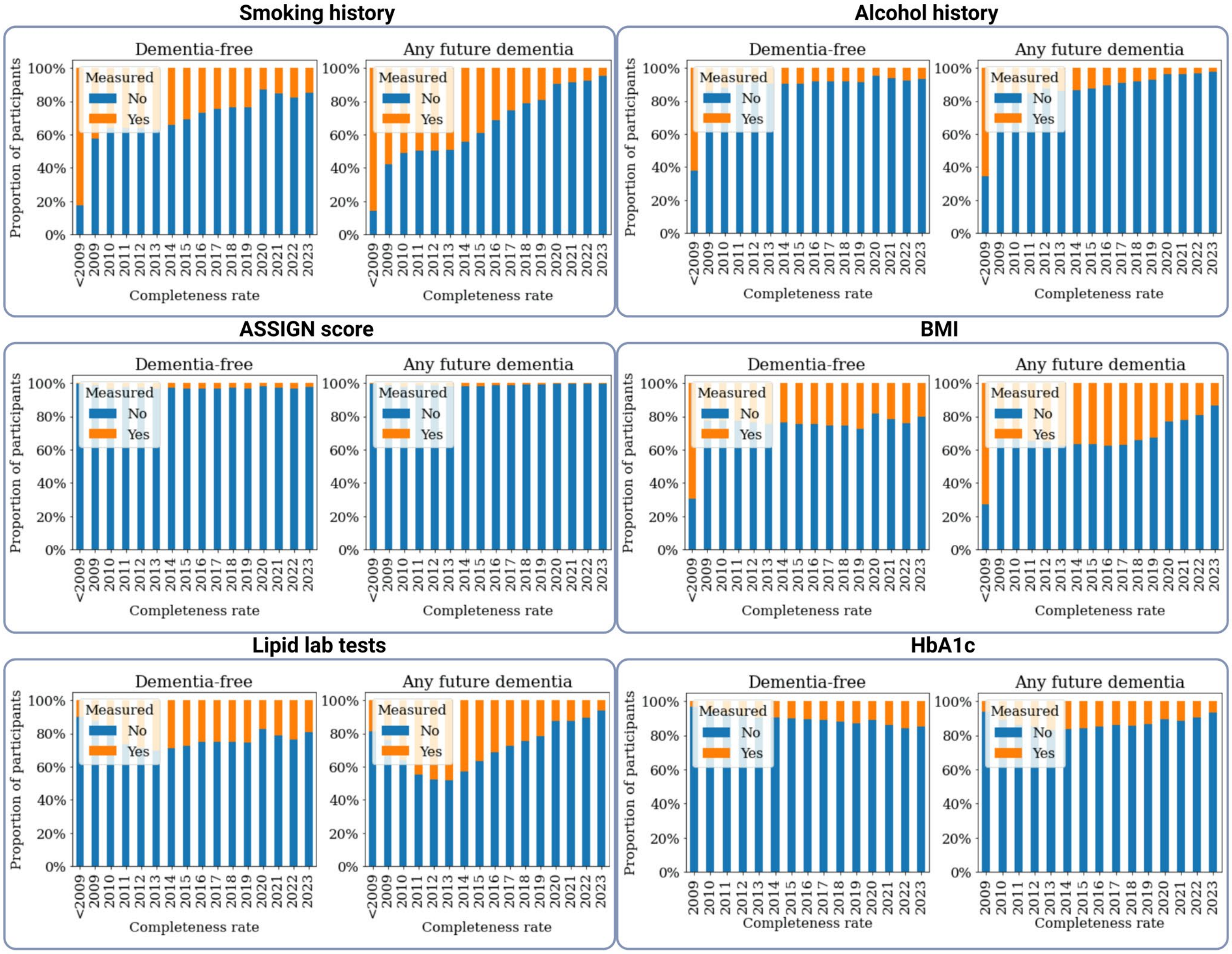

Supplementary Fig. 1. Annual distribution of missingness across some of the clinically-curated data variables for predicting dementia incidence. Generally, rate of completeness is higher in the dementia cohort in the early stages of the prediction window, while, the reverse is true in the final years of observation. Lipids group include: Total Cholesterol, HDL/LDL Cholesterol, Total to HDL Cholesterol Ratio and Triglyceride tests. BMI – Body mass index, ASSIGN - cardiovascular risk calculator validated in Scottish populations (Scottish Heart Health Extended Cohort). Sample sizes: Smoking status (n=122,885 measured in dementia-free group, n=11,063 measured in dementia group), Alcohol intake (n=101,362 measured in dementia-free group, n=9,038 measured in dementia group), ASSIGN score (n=34,149 measured in dementia-free group, n=1,432 measured in dementia group), BMI (n=115,217 measured in dementia-free group, n=10,870 measured in dementia group), Lipid tests (n=91,491 measured in dementia-free group, n=9,920 measured in dementia group), HbA1c test (n=52,441 measured in dementia-free group, n=5,453 measured in dementia group).

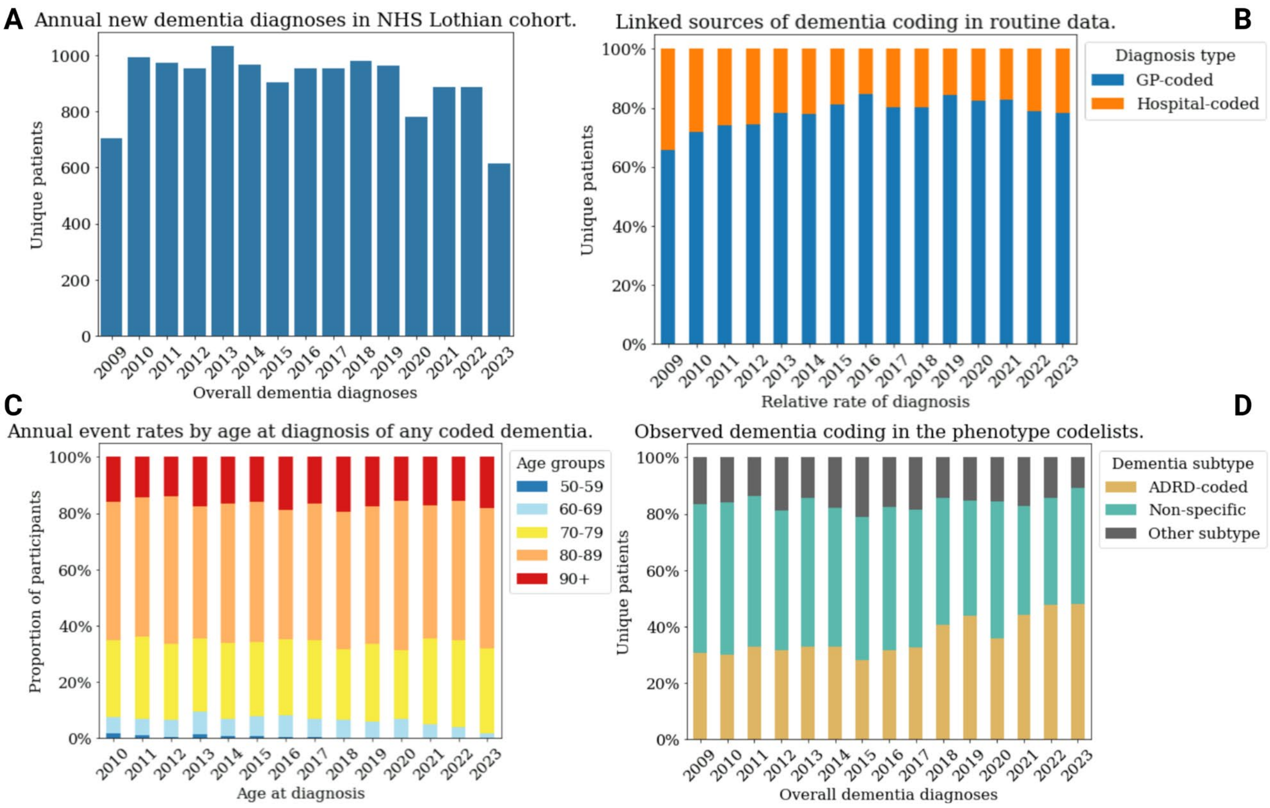

Supplementary Fig. 2. The annual distribution of coded dementia diagnoses in unique patients from the processed NHS Lothian cohort. (A) - the annual distribution of coded diagnoses. (B) – annual relative frequency by source of diagnosis (n=1,854 for overall patients with GP-coded diagnosis; n=602 for overall patients with Hospital-coded diagnosis). (C) - Observed annual event rates at the age of diagnosis (n=53 patients in 50-59 group; n=703 patients in 60-69 group; n=3,148 patients in 70-79 group; n=5,633 in 80-89 group; n=1,906 in 90+ group). (D) Annual distribution across specificity of dementia coding (n=4,855 for ADRD; n=6,481 for non-specific dementia subtypes; n=2,196 for other specified subtypes). ADRD – Alzheimer's disease coded dementia phenotype.

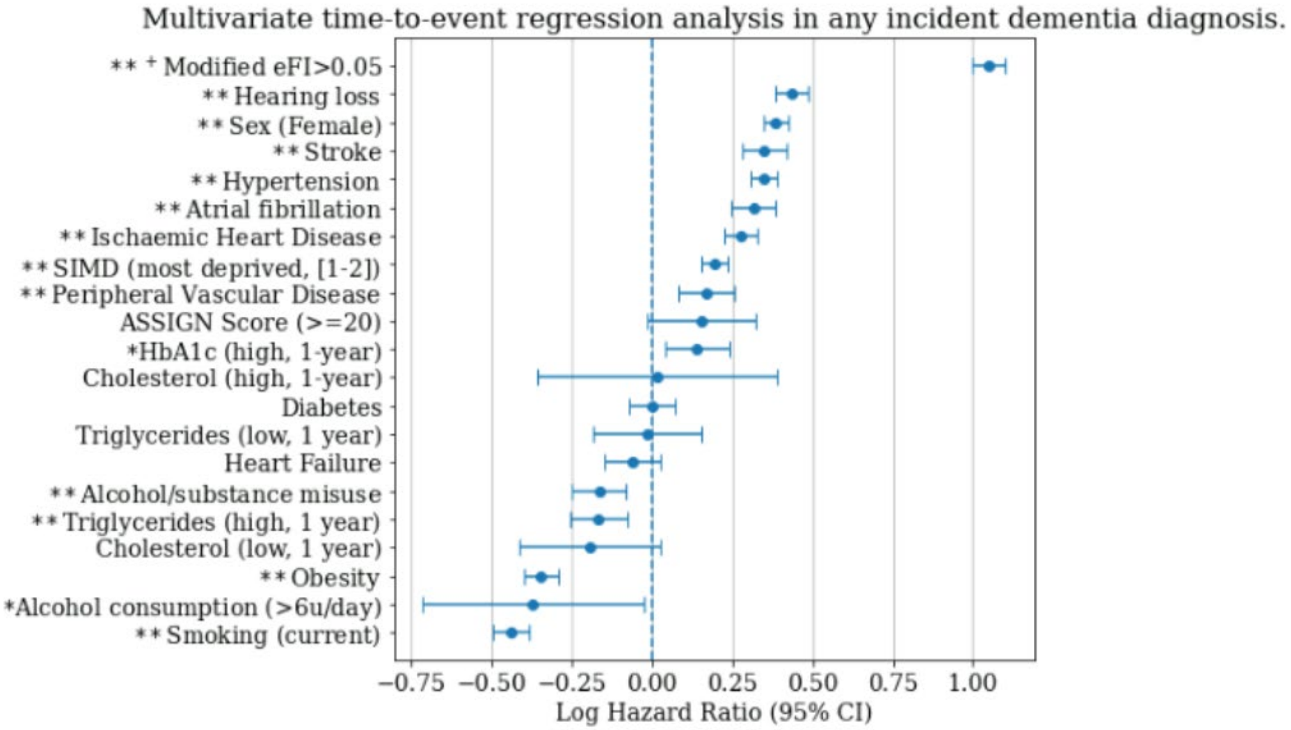

Supplementary Fig. 3. Forest plot showing the log-transformed hazard ratio (95% CI) estimates in a multivariate Cox regression model for incidence of dementia using the clinically-supervised feature set (excluding age). Analysis is based on months to diagnosis event and features are sorted by the log-hazard ratio in descending order. Cutoff for the eFI score was selected based on the whole population median. The significance of individual regression coefficients was tested using the Wald statistic: ** - p<0.005; * - p<0.05. Coefficients and sample size: Modified eFI>0.05 (n=69,809; z=39.87; p<0.005), Hearing loss (n=10,293; z=16.14; p<0.005), Female (n=75,274; z=19.54; p<0.05), Stroke (n=5,464; z=10.15; p<0.005), Hypertension (n=48,117; z=16.18; p<0.005), Atrial fibrillation (n=6,447; z=9.23; p<0.005), Ischaemic Heart Disease (n=13,655; z=10.33; p<0.005), SIMD quintiles 1-2 (n=35,264; z=9.39; p<0.005), Peripheral Vascular Disease (n=3,692; z=3.74; p<0.005), ASSIGN Score >=20 (n=1,178; z=1.77; p=0.08), High HbA1c at 1 year (n=4,878; z=2.71; p<0.05), High Total Cholesterol at 1 year (n=231; z=0.08; p=0.94), Diabetes (n=12,278; z=0.03; p=0.97), Low Triglycerides at 1 year (n=1,099; z=-0.19; p=0.85), Heart failure (n=4,305; z=-1.43; p=0.15), Alcohol or substance misuse (n=8,176; z=-3.77; p<0.005), High Triglycerides at 1 year (n=5,169; z=-3.63; p<0.005), Low Total Cholesterol at 1 year (n=660; z=-1.73; p=0.08), Obesity (n=18,266; z=-12.26; p<0.005), High alcohol consumption (n=828; z=-2.12, p<0.05), Current smoker (n=26,303, z=-15.31, p<0.005). SIMD – Scottish Index for Multiple Deprivation. eFI – modified electronic Frailty Index. ASSIGN - cardiovascular risk calculator validated in Scottish populations (Scottish Heart Health Extended Cohort).

[+] Excludes deficits coded as 'Memory & Cognitive problems' from the estimation.

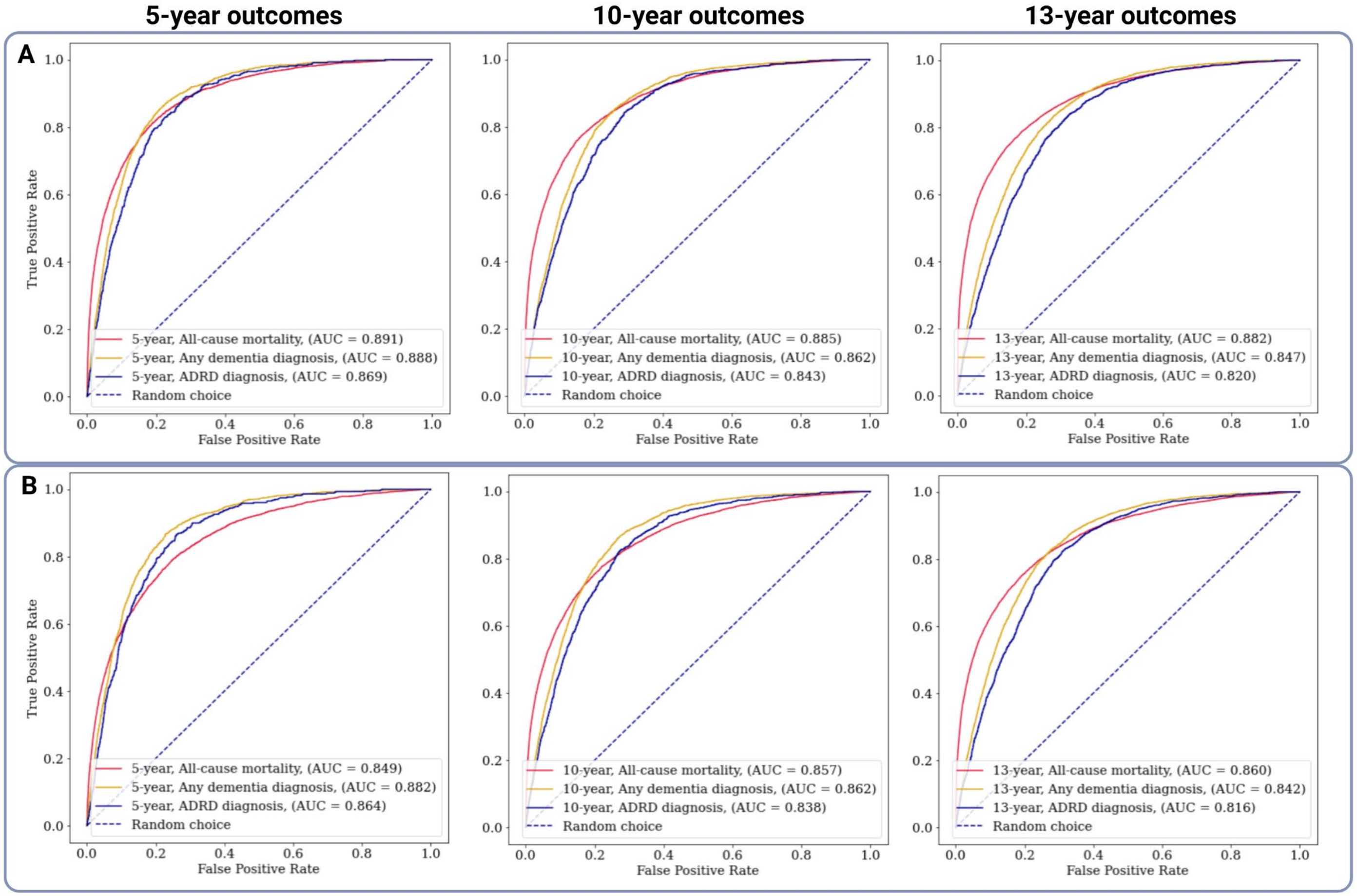

Supplementary Fig. 4. Receiver Operating Characteristic (ROC) curves showing the calibrated discrimination performance (Area under-the-curve [AUC]) in non-specific coded dementia, ADRD (Alzheimer's disease-related dementia) and all-cause mortality. Models in (A) include the data-driven feature set, while models shown in (B) include the predictive performance on the clinically-supervised set. The overall prevalence of dementia was n=1,300 (3%) in any diagnosis, n=416 (1%) in ADRD diagnosis, n=4,032 (9%) in all-cause mortality for 5-year outcomes; n=2,669 (6%) in any diagnosis, n=936 (2%) in ADRD diagnosis, n=8,761 (20%) in all-cause mortality for 10-year outcomes; n=3,433 (8%) in any diagnosis, n=1,235 (3%) in ADRD diagnosis, n=12,022 (28%) in all-cause death for 13-year outcomes.

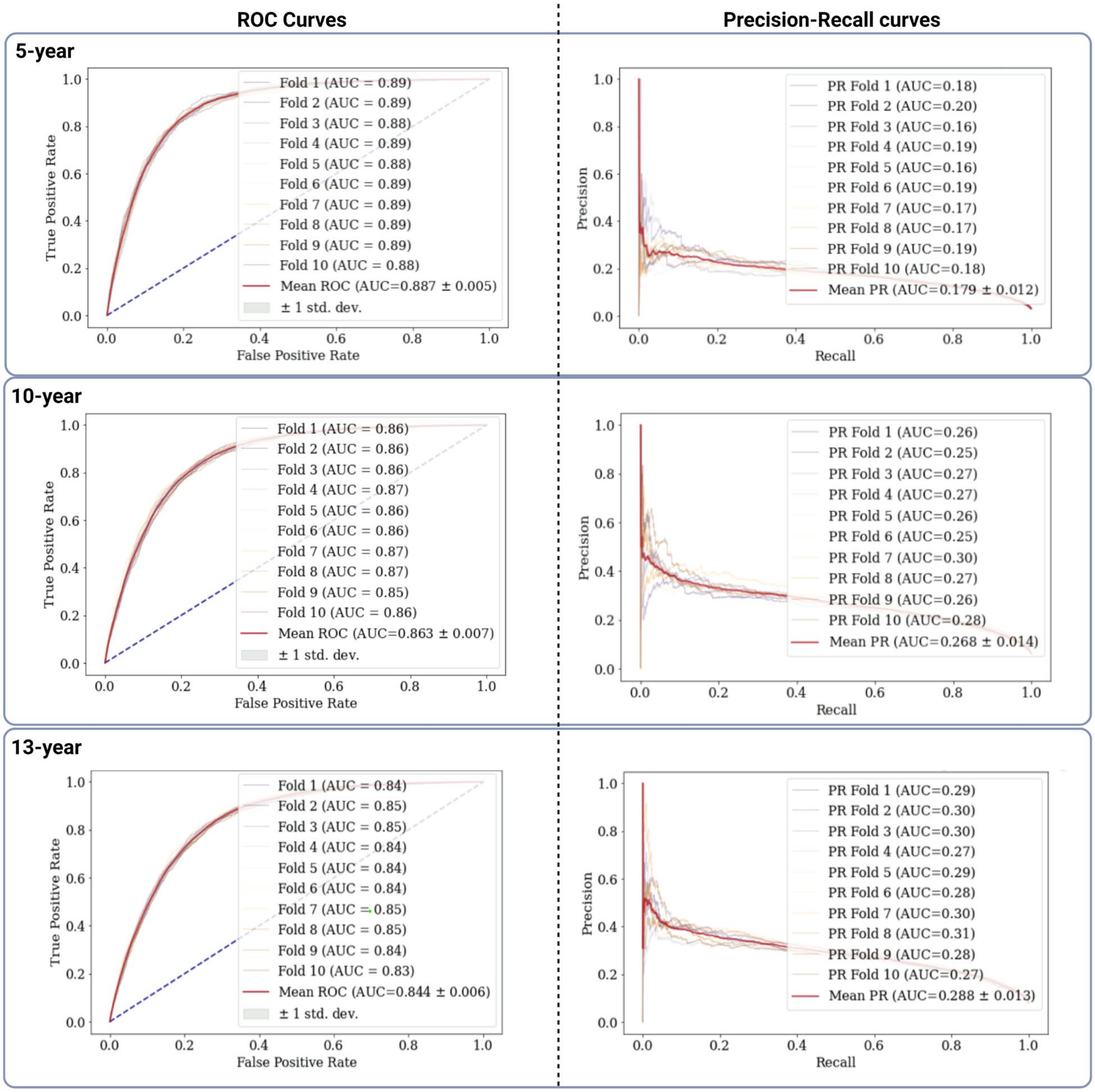

Supplementary Fig. 5. Stratified 10-fold cross-validation results for predicting any incident dementia across each target window. The model performs stable on random partitions over the complete population across ROC and Precision-Recall curves. Cross-validation was performed on the clinically-supervised model subsets, achieving comparable performance to the measures evaluated on the hold-out validation set. Sample size: n=129,701 in Folds 1-3 and 129,702 in Folds 4-10. The overall prevalence of dementia was n=1,300 (3%) for 5-year outcomes, n=2,669 (6%) for 10-year outcomes and n=3,433 (8%) for 13-year outcomes.

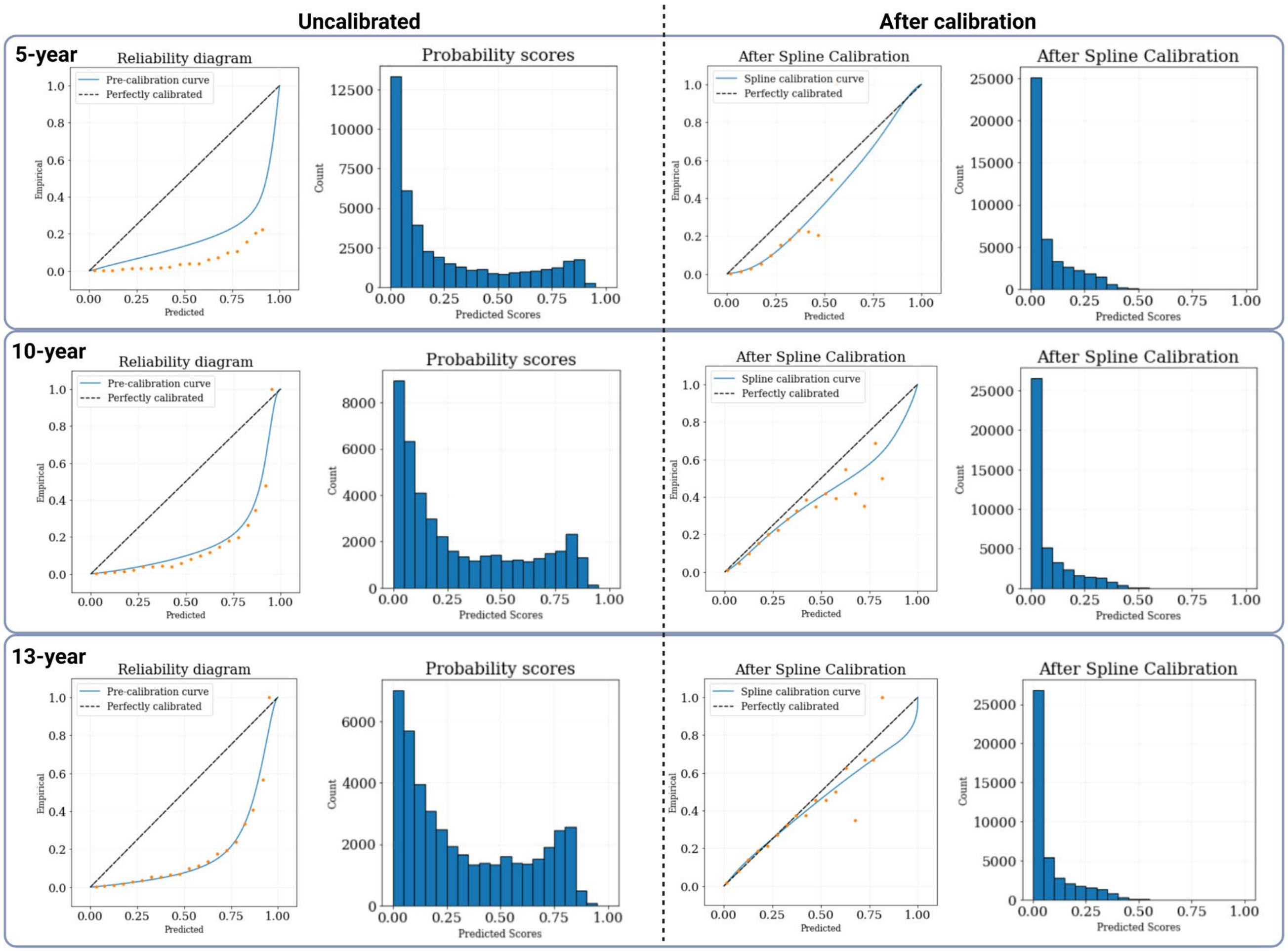

Supplementary Fig. 6. Calibration curves detailing the relationships between the model probability scores and the actual event rate of dementia, as well as the absolute probability distributions before and after the calibration. Calibration was performed using the Cubic Spline method fitted on a random stratified split, holding out 30% of the remaining training set (after the initial train/validation split) during the evaluation stage. The calibration curves were then examined using the same validation set used in the rest of the evaluation procedures. The model probabilities were stratified into equally-spaced bins. Each data point on the calibration curve corresponds to the average predicted probability across each bin and its corresponding observed probability in the validation set. The model is better calibrated when the binned data point is closer to the diagonal. The calibration curves shown were generated using the data-driven feature subset. Samples in calibration set: n=30,264, Samples in validation set: n=43,234.

Model performance over different Machine Learning classifiers (13-year dementia diagnosis outcome).

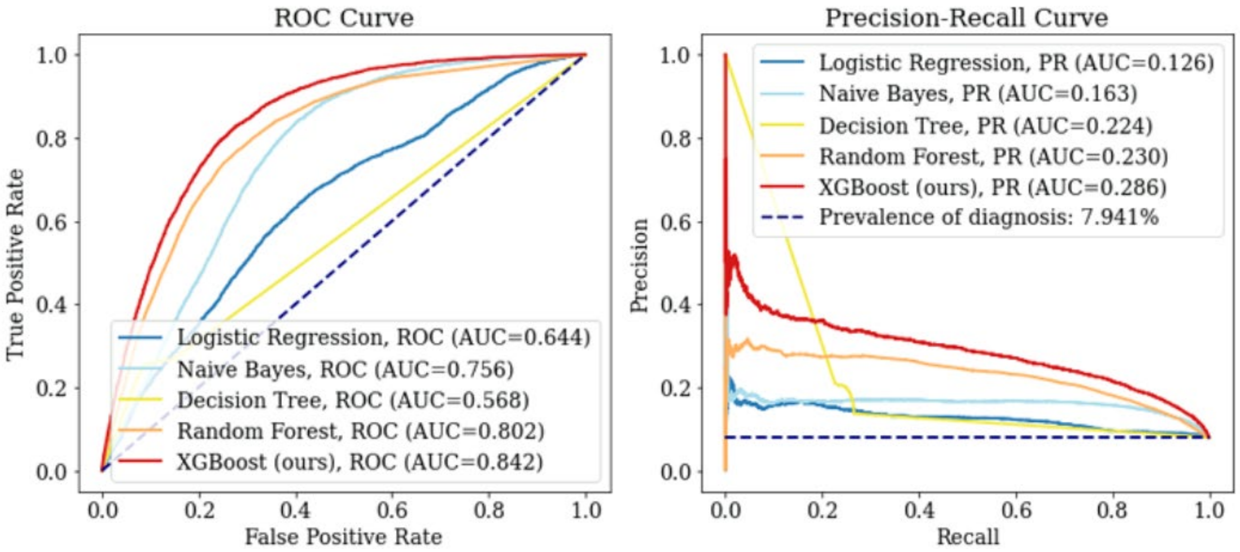

Supplementary Fig. 7. Performance comparison across the Receiver Operating Characteristic (ROC) and Precision-Recall area-under-the-curves in various commonly adapted Machine Learning classifiers. This simulation was performed over the clinically-supervised feature subset for predicting 13-year outcomes of future dementia diagnosis.

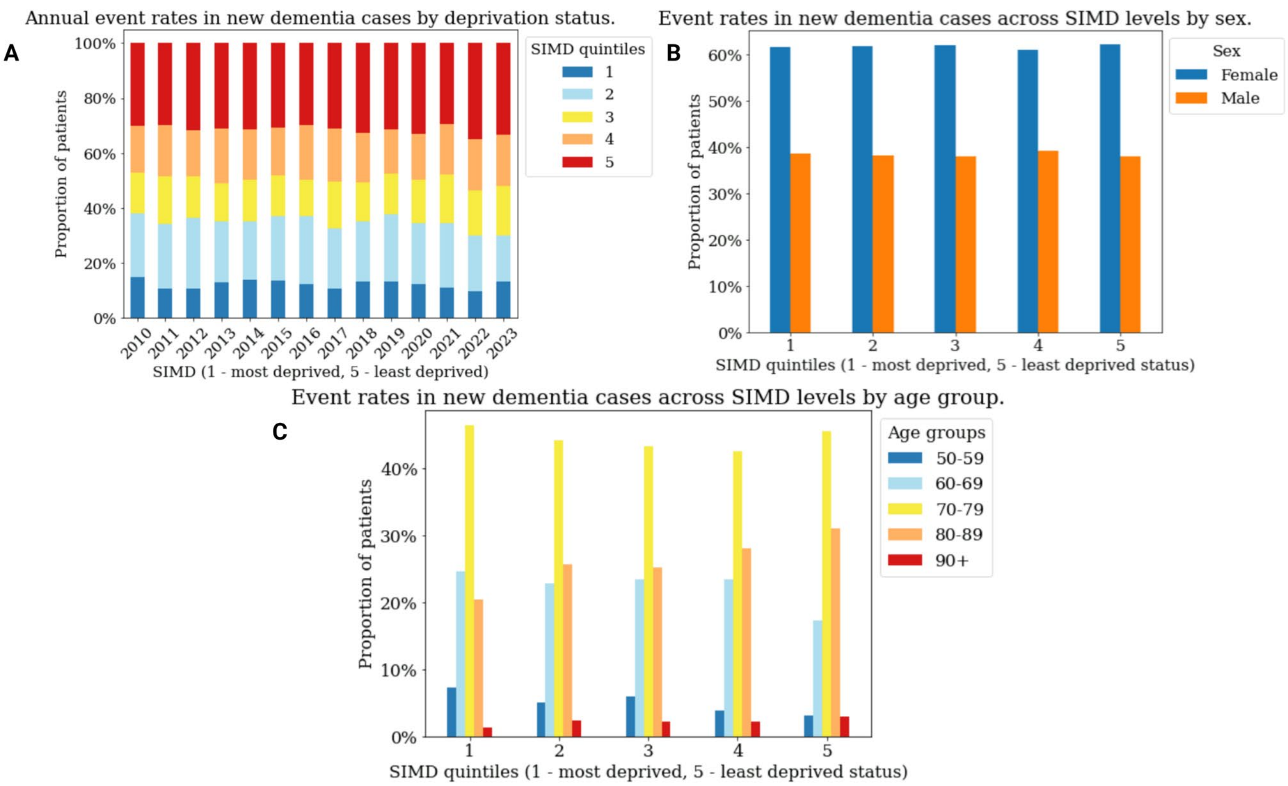

Supplementary Fig. 8. Exploratory data analysis detailing inequalities by deprivation status in patients with dementia incidence. (A) – annual relative distribution of deprivation quintiles in the dementia population (n=1,256 in group 1 with highest deprivation; n=2,362 in group 2; n=1,597 in group 3; n=1,872 in group 4; n=3,245 in group 5 with lowest deprivation). B – gender distribution across each deprivation quintile in the dementia group (Quintile 1: n=773 in females, n=483 in males; Quintile 2: n=1,459 in females, n=903 in males; Quintile 3: n=990 in females, n=607 in males; Quintile 4: n=1,139 in females, n=733 in males; Quintile 5: n=2,015 in females, n=1,230 in males). C – variation in age across each deprivation quintile in the dementia group (Quintile 1: n=92 for age 50-59, n=309 for age 60-69, n=582 for age 70-79, n=256 for age 80-89, n=17 for age 90+; Quintile 2: n=119 for age 50-59, n=537 for age 60-69, n=1,044 for age 70-79, n=607 for age 80-89, n=55 for age 90+; Quintile 3: n=96 for age 50-59, n=374 for age 60-69, n=691 for age 70-79, n=401 for age 80-89, n=35 for age 90+; Quintile 4: n=73 for age 50-59, n=438 for age 60-69, n=795 for age 70-79, n=524 for age 80-89, n=42 for age 90+; Quintile 5: n=103 for age 50-59, n=562 for age 60-69, n=1,475 for age 70-79, n=1,008 for age 80-89, n=97 for age 90+). SIMD – Scottish Index of Multiple Deprivation.

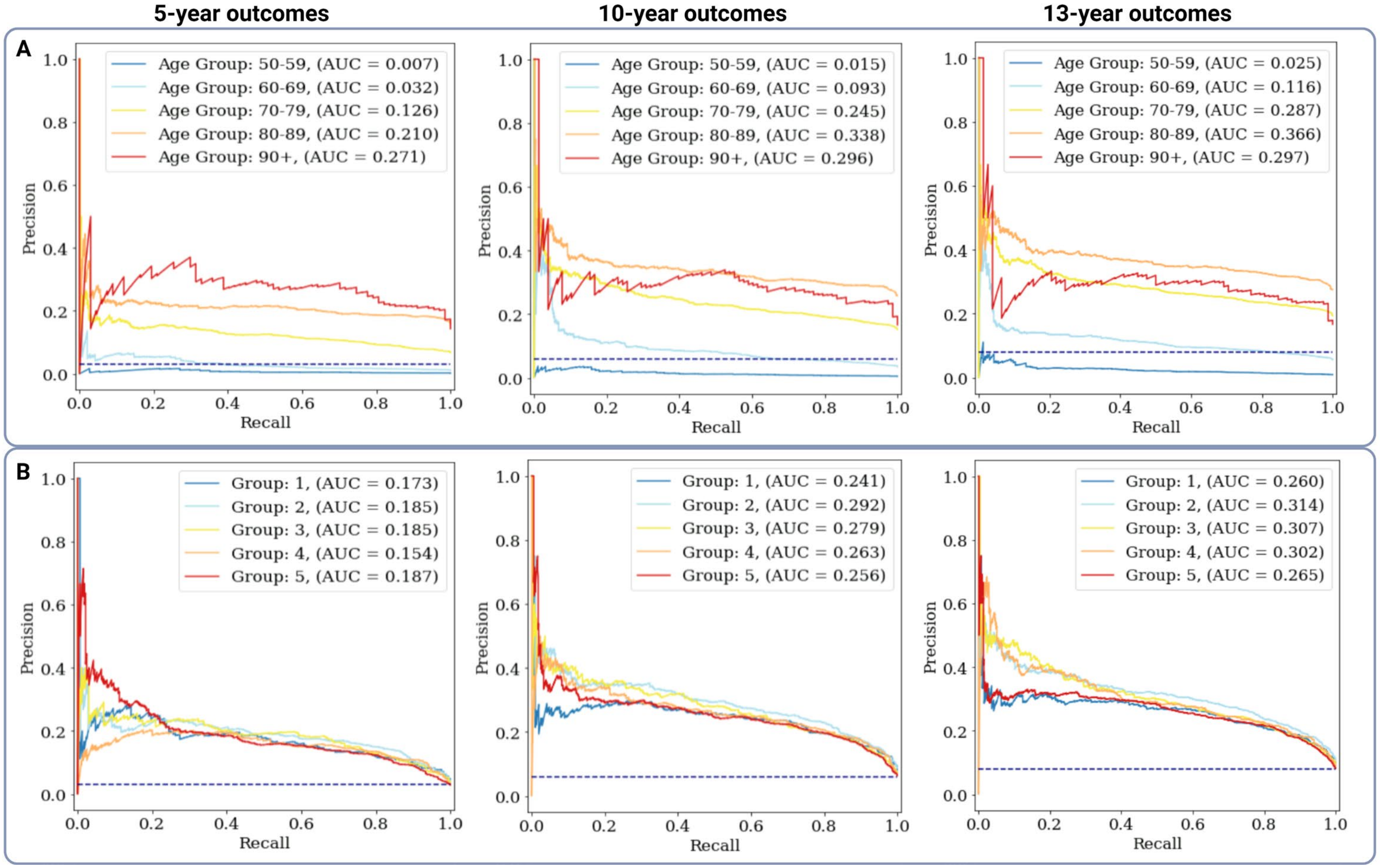

Supplementary Fig. 9. Stratified analysis results comparing the Precision-Recall curves across age and socioeconomic deprivation groups on the clinically-supervised feature subset. (A) - performance across the defined age groups across the 5, 10-year and any diagnosis outcomes (Sample size: n=18,352 in 50-59 group; n=13,170 in 60-69 group; n=7,867 in 70-79 group; n=3,377 in 80-89 group; n=468 in 90+ group). (B) - performance across each SIMD quintile (Sample size: n=3,849 in Quintile 1; n=6,694 in Quintile 2; n=5,112 in Quintile 3; n=6,168 in Quintile 4; n=11,686 in Quintile 5). Horizontal dashed lines represent the overall prevalence of dementia in the respective model subset (n=1,300 (3%) for 5-year outcomes, n=2,669 (6%) for 10-year outcomes and n=3,433 (8%) for 13-year outcomes). SIMD – Scottish Index for Multiple Deprivation (1 – most deprived, 5 – least deprived).
